# Bodily Sensory Inputs and Anomalous Bodily Experiences in Complex Regional Pain Syndrome: Evaluation of the Potential Effects of Sound Feedback

**DOI:** 10.3389/fnhum.2017.00379

**Published:** 2017-07-27

**Authors:** Ana Tajadura-Jiménez, Helen Cohen, Nadia Bianchi-Berthouze

**Affiliations:** ^1^UCL Interaction Centre, University College London London, United Kingdom; ^2^Department of Psychology, Universidad Loyola Andalucía Seville, Spain; ^3^Human Neuroscience Lab, Universidad Loyola Andalucía Seville, Spain; ^4^Division of Medicine, University College London London, United Kingdom; ^5^Rheumatology, Pain & Rehabilitation, Royal National Orthopaedic Hospital Stanmore, United Kingdom

**Keywords:** body perception, body representation, anomalous bodily experiences, complex regional pain syndrome, action sounds, body-related sensory inputs, multisensory interaction, technologies for self-management

## Abstract

Neuroscientific studies have shown that human's mental body representations are not fixed but are constantly updated through sensory feedback, including sound feedback. This suggests potential new therapeutic sensory approaches for patients experiencing body-perception disturbances (BPD). BPD can occur in association with chronic pain, for example in Complex Regional Pain Syndrome (CRPS). BPD often impacts on emotional, social, and motor functioning. Here we present the results from a proof-of-principle pilot study investigating the potential value of using sound feedback for altering BPD and its related emotional state and motor behavior in those with CRPS. We build on previous findings that real-time alteration of the sounds produced by walking can alter healthy people's perception of their own body size, while also resulting in more active gait patterns and a more positive emotional state. In the present study we quantified the emotional state, BPD, pain levels and gait of twelve people with CRPS Type 1, who were exposed to real-time alteration of their walking sounds. Results confirm previous reports of the complexity of the BPD linked to CRPS, as participants could be classified into four BPD subgroups according to how they mentally visualize their body. Further, results suggest that sound feedback may affect the perceived size of the CRPS affected limb and the pain experienced, but that the effects may differ according to the type of BPD. Sound feedback affected CRPS descriptors and other bodily feelings and emotions including feelings of emotional dominance, limb detachment, position awareness, attention and negative feelings toward the limb. Gait also varied with sound feedback, affecting the foot contact time with the ground in a way consistent with experienced changes in body weight. Although, findings from this small pilot study should be interpreted with caution, they suggest potential applications for regenerating BDP and its related bodily feelings in a clinical setting for patients with chronic pain and BPD.

## Introduction

Anomalous bodily experiences accompany a number of chronic pain conditions, such as in the case of complex regional pain syndrome (CRPS), also known as Reflex Sympathetic Dystrophy Syndrome (RSD). CRPS may initially affect a single limb, but rarely may then spread throughout the body. It may occur following injury and major nerve damage (Type 2), or after minor trauma with no apparent nerve injury, or spontaneously (Type 1). The cause of CRPS is unclear and is likely to involve multiple different mechanisms involving inflammation, the immune system, and the autonomic, peripheral and central nervous systems (Rockett, 2014). The incidence of CRPS type 1 varies from 5.46 per 100,000 person-years at risk with a prevalence of 20.57 per 100,000 (Sandroni et al., 2003), to 26.2 per 100,000 person-years (de Mos et al., 2007). Sufferers of CRPS describe a severe, continuous, and debilitating pain in their affected limb, and 55–85% of these sufferers experience some sort of body perception disturbances (Lewis and McCabe, 2010).

**Figure 1:**
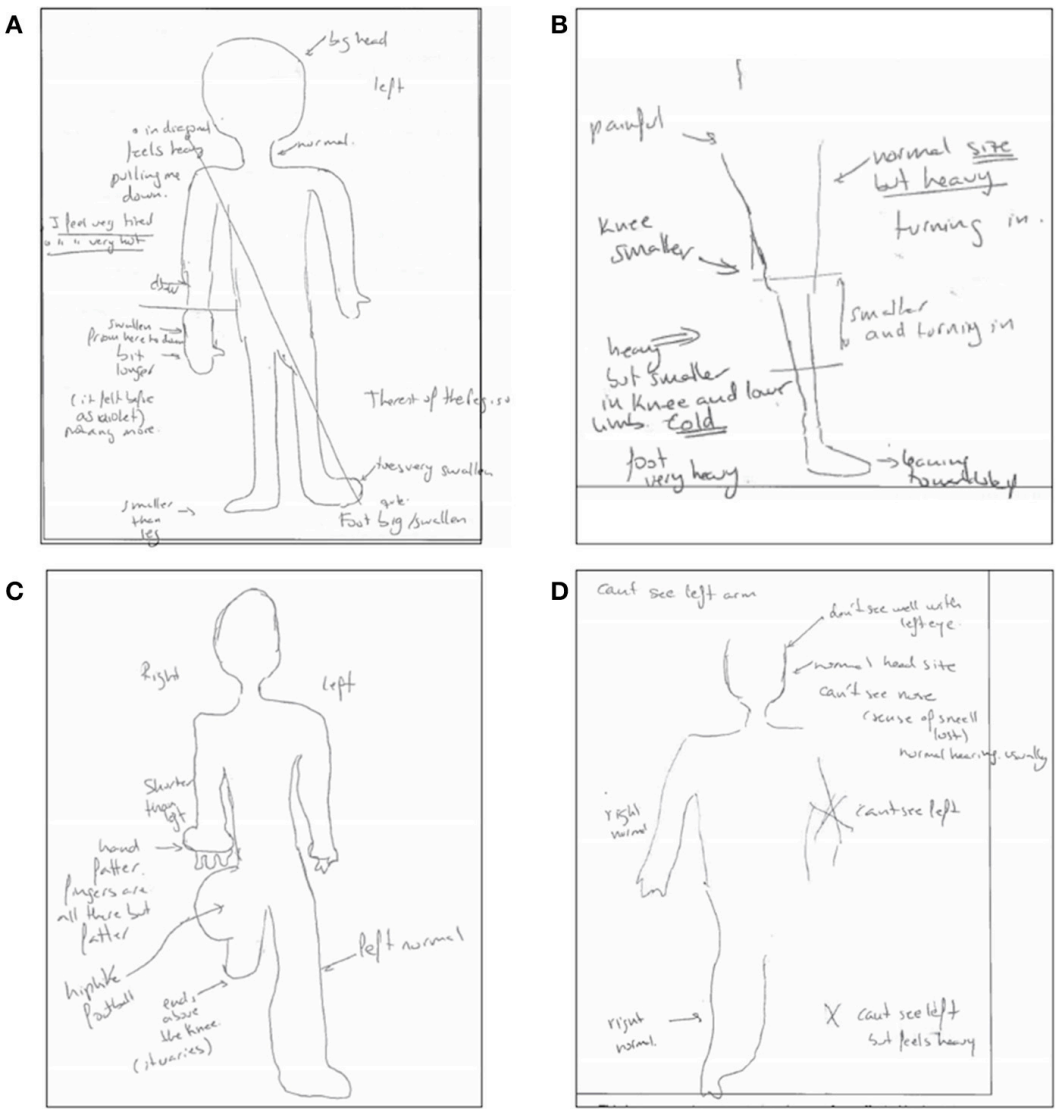
Examples of anomalous bodily experiences in CRPS. The Figure displays four drawings generated from descriptions provided by participants when asked to visualize their body with eyes closed as part of the Bath CRPS Body Perception Disturbance Scale. Notes on the drawing read: **(A)** “big head,” “feels heavy, pulling me down,” “I feel very tired,” “I feel very hot,” “swollen from here to down and a bit longer. Before it felt as violet” [referring to the lower part of the right arm], “foot is big and swollen, toe is very swollen” [referring to the left foot], “the rest of the leg is ok”; **(B)** “painful,” “heavy but smaller in knee and lower limb; turning in and cold,” “foot very heavy and leaning toward left,” “normal size but heavy; turning in” [referring to the rest of the leg]; **(C)** “shorter than left, hand fatter, fingers are all there but fatter” [referring to right arm], “hip like a football,” “ends above the knee” [referring to right leg]; “left side is normal”; **(D)** “can't see the left arm,” “don't see well with left eye,” “can't see the nose (the sense of smell is lost, hearing is usually normal), “can't see left leg, but feels heavy,” “right side is normal.”

They describe abnormal sensations such as segments of their limb being perceived as being much larger, heavier, or different in shape, temperature, or pressure from objective assessment; sometimes sections of the limb may also be reported as being missing during mental visualization (some examples are given in Figure 1; Moseley, 2005; Lewis and McCabe, 2010; Turton et al., 2013). Other disturbances include a sense of disowning the affected limb or difficulties in moving it; lack of awareness as to the position of the impaired limb; and hostile feelings toward the limb, such as hate, anger, disgust, or repulsion, that often lead to a desire to amputate this limb (Galer and Jensen, 1999; Förderreuther et al., 2004; Lewis et al., 2007; Harden et al., 2010; Lewis and McCabe, 2010). Furthermore, significant motor dysfunction is a common symptom in people with CRPS (Galer et al., 1999). CRPS is a deeply distressing condition that has a significant impact on the sufferer's quality of life. A significant number experience lasting symptoms, and some experience chronic pain and disability (Bean et al., 2014). There is currently no cure for this condition, and pain may continue indefinitely despite treatment attempts. While CRPS affects far fewer people than other chronic pain conditions such as fibromyalgia, patients with CRPS may present extreme body-perception disturbances (BPD) and thus CRPS becomes a good model condition to study anomalous bodily experiences.

Previous works have described that some people with CRPS have referred sensations (i.e., the perception of a stimulus at a location distant from the stimulated body site; McCabe et al., 2003). These sensations are thought to be a clinical correlate of the cortical reorganization found in neuroimaging, psychophysical and transcranial magnetic stimulation studies in areas of the primary and secondary somatosensory cortex responsible for the representation of the affected limb (Flor et al., 1995, 1997; Maihöfner et al., 2003, 2004; McCabe et al., 2003; Eisenberg et al., 2005; Pleger et al., 2005; Marinus et al., 2011). There is also clinical evidence that in people with CRPS there can be dysfunction of parietal regions (Schwoebel et al., 2001; Cohen et al., 2013). These regions overlap with multisensory parietal areas integrating somatosensory, visual and auditory signals to form mental body representations (Serino et al., 2013).

The term body-representation refers to the internal knowledge of the size and shape of one's body parts and its position in space relative to each other (Longo et al., 2010; Medina and Coslett, 2010). Body-representations are not only essential for everyday motor functioning, but are also tightly linked to emotional processes (Carruthers, 2008; Pollatos et al., 2008). As demonstrated by neuroscientific and psychological research, body-representations are continuously updated by multisensory information received during bodily interactions with the environment (Tsakiris, 2010). Indeed, whereas one's body does not often change appearance very quickly, the mental representation of body appearance can update very quickly in response to sensory feedback (Botvinick and Cohen, 1998; Maravita and Iriki, 2004; Serino and Haggard, 2010; Longo and Haggard, 2012). There are now numerous examples of artificial manipulations of body-representations using specially designed sensory feedback. For instance, in the so-called *rubber hand* illusion, people experience an artificial rubber hand as their own if they see the rubber hand being touched and in synchrony they feel their own hand being touched (Botvinick and Cohen, 1998). A related illusion is the so-called *body swap* illusion, in which people experience an entire artificial body as their own if they are administered tactile stimulation on their chest while they observe a synchronous touch on a manikin's chest (Petkova and Ehrsson, 2008; van der Hoort et al., 2011). These changes in body-representation are triggered by integration of discrepant visual, tactile, and proprioceptive information. A few recent studies have shown that sound can also be used for inducing changes in the perceived physical appearance of one's own body and that these changes have an effect in the related emotional state and patterns of bodily movement (Tajadura-Jiménez et al., 2012, 2015a,b, 2016). We highlight one of these studies in which we showed that the altering of walking sounds to make them consistent with those produced by a lighter body leads to represent one's body as slimmer, as well as enhancing emotional state and changing gait biomechanics in a way consistent with having a lighter body (Tajadura-Jiménez et al., 2015a).

The above studies show that sensory inputs are responsible for forming and updating mental body-representation. Of relevance to the present research, some of these studies have proposed that the altering of sensory cues related to one's body can result in reorganization within the somatosensory cortex (Taylor-Clarke et al., 2004; de Vignemont et al., 2005; Haggard et al., 2007; Cardinali et al., 2009, 2012; Cardini et al., 2011, 2012, 2013; Tajadura-Jiménez et al., 2012, 2016; Canzoneri et al., 2013a,b; Miller et al., 2014; Cardini and Longo, 2016). In relation to people with CRPS, it has been suggested that the lack of sensory input from the limb may contribute to the perpetuation of their BPD, as they are often reluctant to look at or touch their affected limb, choosing to position it in such a way that it is outside their field of view and even trying to avoid thinking about it (Lewis et al., 2007; Lewis and McCabe, 2010).

Treatment for CRPS often utilizes a combination of cognitive strategies which encourage patients to visualize their affected limb and think about it in a positive way, and sensory-motor strategies encouraging them to move, look and touch the limb to provide accurate sensory inputs that help correct the BPD. Other sensory therapies, known as sensory discrimination training or desensitization therapy involve subjecting the limb to a range of textures and other stimuli such as thermal challenges (Moseley, 2008; Lewis and McCabe, 2010). Such approaches are recommended in therapeutic guidelines for CRPS (Goebel et al., 2012) and they are a core component of multi-disciplinary rehabilitation programs, but there is little published evidence to support this practice (Stanton-Hicks et al., 1998, 2002).

Some people with CRPS may find sensory interventions involving looking at or touching their affected limb upsetting for them, given the previously mentioned reluctance to look at or touch the limb (Lewis et al., 2007; Lewis and McCabe, 2010). In some of these cases mirror-visual feedback may become a useful aid in CRPS rehabilitation because it avoids direct contact with the affected limb, yet it provides visual inputs that help updating limb representations (Lewis and McCabe, 2010). Here, we explored for the first time the possibility of using sound-feedback to help with regenerating distorted mental body-representations in people with CRPS. The use of sound feedback in this case offers a number of interesting advantages, as apart from removing the need for direct visual contact with the affected limb, it can provide a continuous flow of information, as audition never “turns off” in the same way that vision is blocked when shutting our eyes, and it does not interfere with movement. Further, for the specific design of the sound feedback, we built on our previous findings on healthy people that real-time alteration of self-produced walking sounds can alter people's perceptions of their body size/weight, while enhancing gait patterns and people's positivity toward their bodies (Tajadura-Jiménez et al., 2015a). Of relevance, other recent studies from our group have demonstrated that real-time sound-feedback on one's movement can be used for sensory substitution of defective proprioception in people with low back pain, increasing confidence and motivation for physical activity in these populations (Singh et al., 2014, 2016).

While it has been demonstrated that sound can alter body perception in healthy controls, it is unknown whether this is possible in the context of chronic pain and BPD. The aim of this proof-of-principle pilot study was to establish whether sound can be used to alter BPD in CRPS. The hypothesis was that the altering of the auditory feedback derived from one's footsteps would lead to an enhanced perception of one's body and its related emotional state and gait in those with CRPS. To date this approach has not been trialed in CRPS. The findings may help to ascertain the feasibility and potential value of auditory simulation for regenerating BPD and its related bodily feelings in a clinical setting.

## Materials and methods

### Participants

Twelve participants were recruited (10 female and 2 male; mean age ± *SD*: 49.0 ± 8.4 years; age range from 36 to 64 years—see individual demographic characteristics of participants in Table 1). The inclusion criteria were the following: (1) age comprised between 18 and 70 years old; (2) meet the recognized diagnostic research criteria for CRPS Type I; and (3) able to walk continuously for at least 60 s with or without walking aids. The exclusion criteria were the following: (1) diagnosis of any other neurological, psychopathologic, motor disorder, or major nerve damage (CRPS Type II); (2) disability significantly affecting physical mobility/activity; (3) the presence of any other limb pathology or pain on the affected CRPS limb; (4) hearing impairment; (5) weight <40 kg or more than 135 kg; (6) severe Postural Orthostatic Tachycardia Syndrome (POTS); (7) insufficient mental capacity to take part in the study; and (8) unable to understand written or verbal English and give informed consent. The characteristics of each participant, including demographics, duration of CRPS condition and body part affected are listed in Table 1.

**Table 1:** Demographic characteristics of participants with CRPS.

**Gender**	**Age at experiment**	**Duration CRPS (years and months)**	**Body part affected**	**BPD group**
Female	40	1 year and 6 months	Right lower limb	Big
Female	48	1 year and 9 months	Right upper limb/left lower limb	Big
Female	62	3 years and 2 months	Right upper limb	Big
Female	49	8 years	Right lower limb	Mixed
Female	56	3 years and 7 months	Right lower limb	Mixed
Female	46	4 years and 2 months	Right lower limb	Small
Male	36	1 year	Right lower limb	Nothing
Female	39	17 years and 4 months	Left upper limb	Nothing
Male	64	4 years and 5 months	Both lower limbs	Nothing
Female	52	9 years	Both lower limbs (left worse than right)	Nothing
Female	52	16 years	Both upper limbs (possibly left lower limb)	Nothing
Female	44	1 year and 8 months	Left lower limb	Nothing

Participants were recruited through the Royal National Orthopaedic Hospital (RNOH) at Stanmore from a tertiary referral service for those with CRPS. Potential participants were identified from current patients and from patients who have previously received treatment for CRPS at RNOH and sent an invitation to voluntary take part in the study. Participants were naïve as to the purposes of the study. This study was carried out in accordance with the recommendations of the 1964 Declaration of Helsinki and the ethics committee of the UK National Health Service. All subjects gave written informed consent in accordance with the Declaration of Helsinki. The protocol was approved by the UK National Health Service Research Ethics Committee.

### Apparatus and materials

**Figure 2:**
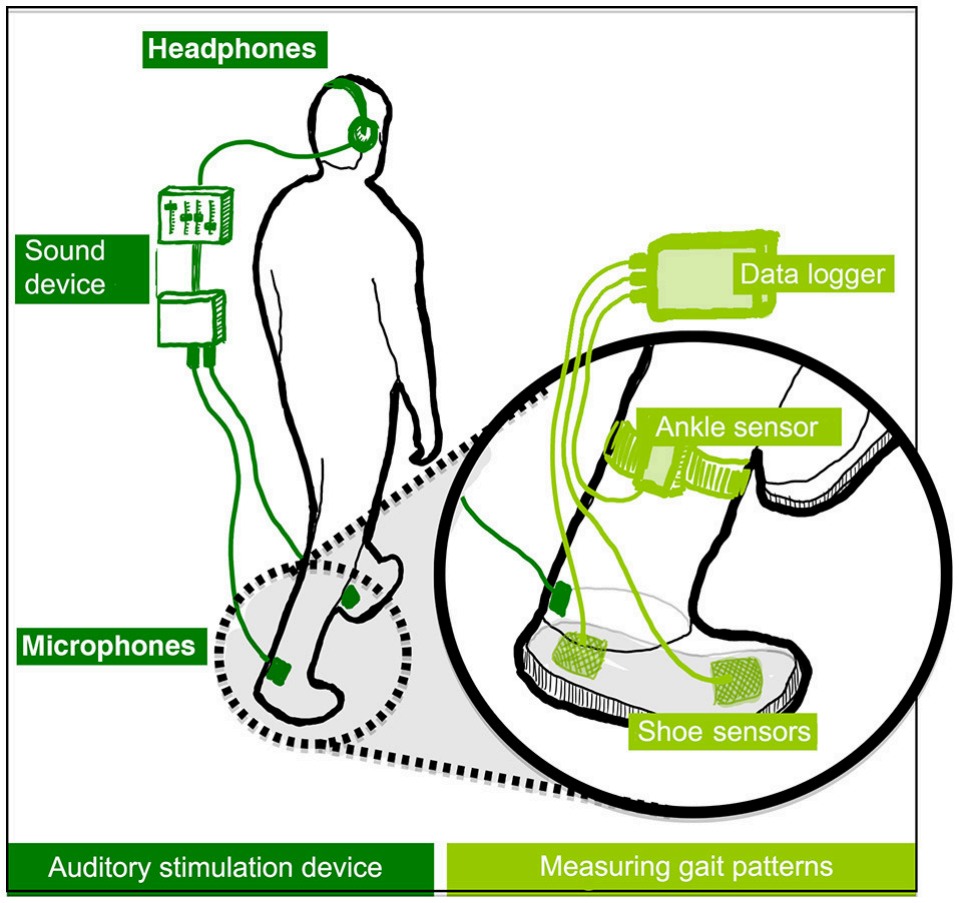
Overview of the auditory stimulation device **(left)** and sensors used for sensing gait **(right)**. In healthy participants, short adaptation periods to altered walking sounds led to lower perceived body weight, to the adoption of gait patterns typical of lighter bodies and to an enhanced emotional state. (Tajadura-Jiménez et al., 2015a), © 2015 ACM, Inc. https://doi.org/10.1145/2702123.2702374. Reprinted by permission. This figure is published in color in the online version.

The experiment was conducted at the local motor learning lab of the RNOH, which is a quiet environment. Participants were asked to walk on the spot (i.e., to imitate the motions of walking, lifting one leg after the other, without actually resulting in any displacement) for short periods of 1 min on the hard rubber platform of a stationary treadmill. This stationary treadmill was used for safety and comfort reasons, as this setting allowed participants to hold onto two parallel bars placed on the sides of the platform. The height of these bars could be adjusted according to the height of participants. A functioning treadmill was not used because early exploratory work had shown that the sound of the treadmill motor interferes with the sound used in the study. During the walking periods participants were asked to wear a system, which is displayed in Figure 2. This system allows the dynamic modification of footstep sounds, as people walk, and measurement of walking behavior changes. Three sound feedback conditions were designed, as described in Section Sound Feedback Conditions. The system was an adaptation of the system used in Tajadura-Jiménez et al. (2015a), with some modifications in the part involved in gait data collection that allowed minimizing the system thus making it easier to carry. The system is comprised by commercial components, including a pair of strap sandals that are easy to wear (EU size 42); two microphones attached to the sandals and that capture the walking sounds (Core Sound, frequency response 20 Hz–20 kHz); and four force-sensitive resistors (FSR; 1.75 × 1.5″ sensing area) attached to the front and the rear part of each sandal insole and that detect the exerted force by feet against the ground (as in Tajadura-Jiménez et al., 2015a). In addition, the device includes two 3-axis accelerometers attached to the participant's ankles (Sparkfun). FSRs and accelerometer in each foot are connected to a Microduino microcontroller board, which combined a Microduino Core, a Microduino Shield Bluetooth 4.0, and a Microduino USBTTL Shield. This board allowed linking the sensors via Bluetooth to a smartphone that acquired their data. The microphones are connected to a small stereo pre-amplifier (SP-24B) and a sound equalizer (Behringer FBQ800) that modify the sound spectra and these connect to a pair of headphones participants wore (Sennheiser HDA 300). These headphones had high passive ambient noise attenuation (>30 dBA) that muffled the actual sound of footsteps. The analog sound loop had minimal latency (<1 ms). Pre-amp and equalizer were fitted into a small backpack the walker could carry (~2 Kg, 35 × 29 × 10 cm).

A 22-inches computer screen, linked to a laptop computer, was placed in front of participants at the edge of the walking platform (~50 cm away from participants), and it was used for the tasks involving estimating body dimensions (see Section Measures). A keypad, placed on the top of one of the parallel bars, was used to collect participants' responses on body estimates. Presentation® software was used to control the stimulus delivery and to record the participant's body estimates.

### Experimental design

#### Sound feedback conditions

Three sound feedback conditions were designed (based on Li et al., 1991; Tajadura-Jiménez et al., 2015a) for the walking periods. These conditions were created by dynamically modifying the footstep sounds people produce as they walk: a “Control” condition in which no sound feedback was provided (headphones were put inside the backpack); a “High frequency” condition in which the frequency components of the footsteps sounds in the range 1–4 kHz were amplified by 12 dB and those in the range 83–250 Hz were attenuated by 12 dB; and a “Low Frequency” condition in which the frequency components in the range 83–250 Hz were amplified by 12 dB and those above 1 kHz were attenuated by 12 dB.

#### Measures

This mixed methods study utilized qualitative and quantitative outcome measures. The effects of sound feedback received during the 1-min walking periods on BPD and the related bodily feelings and patterns of bodily movement were evaluated by combining self-reporting and objective behavioral measures. Specifically, the effects of sound feedback on BPD were measured in three ways: (1) by assessing the effect of sound on perceived body dimensions; (2) by quantifying changes on gait mechanics, as an implicit measure of changes in perceived body weight; and (3) by looking at the effect of sound on CRPS descriptors, pain and other bodily/emotional feelings that may indicate changes in perceived body parts. Data collected included estimates of body dimensions and verbal descriptions of limb perception; questionnaire data on perceived pain and emotional state; and capture of gait data. The measures used are detailed below:

a) Assessment of perceived body dimensions (“avatar,” “aperture,” and “hands” tasks): participants were asked to estimate the size of their affected body part by indicating this size using their two hands (“hands” task). They were also asked to use a line task visualization tool which involved two white vertical lines displayed on the screen on a black background and which could be moved toward each other, or moved further apart, with use of the keypad. With this tool, participants adjusted the distance between the two lines to correspond to the perceived width of their affected body part (“aperture” task; adapted from studies by Linkenauger et al., 2009; Keizer et al., 2013). Participants were also asked to use a body visualization tool (bodyvisualizer.com; used by Tajadura-Jiménez et al., 2015a for the same purpose) in which they adjusted the weight related dimension of the body of a 3D avatar displayed on the screen to correspond to their perceived body size (“avatar” task). Participants' actual weight and the actual dimensions of their entire body and affected body part(s) were recorded as reference.

b) The Short Form McGill Pain Questionnaire (SF-MPQ; Melzack, 1987): This is a self-report questionnaire, which provides a comprehensive assessment of participants' pain. It includes a 0–10 cm visual analog rating scale of pain intensity as well as a comprehensive list of pain descriptors that capture the quality of that pain. Three pain scores are derived from the sum of the intensity rank values of the words chosen for sensory, affective, and total descriptors. This questionnaire is commonly used in pain clinical routine and pain research. Both the SF-MPQ and the longer MPQ from which it is derived have been shown to have good validity (Dubuisson and Melzack, 1976; Wright et al., 2001; Zinke et al., 2010) and reliability (Graham et al., 1980; Strand et al., 2008). The SF-MPQ also includes the Present Pain Intensity (PPI) index of the standard MPQ.

c) Assessment of participants body feelings—The Bath CRPS Body perception Disturbance Scale (referred in this paper as CRPS BPD scale; Lewis and McCabe, 2010) and “questionnaire on body feelings”: The CRPS BPD scale is standardly used in clinical routine with CRPS patients, and includes a set of items and a drawing based on a verbal description of participants' perception of their painful limb with their eyes closed. This is a routine clinical assessment, which is thought to provide an insight into the extent of cortical reorganization (Förderreuther et al., 2004). We quantified other aspects of the experience by asking participants to select a score that best expresses their feelings using 7-point Likert-type response items adapted from previous studies on healthy participants (Tajadura-Jiménez et al., 2015a). It was comprised by 4 statements which range from: “I feel slow” to “I feel quick” (Speed); “I feel light” to “I feel heavy” (Weight); “I feel weak” to “I feel strong” (Strength); “I feel crouched/stooped” to “I feel elongated/extended” (Extension). In addition, in the following four statements participants rated their level of agreement (from “I strongly disagree” to “I strongly agree”): “It seems like the sounds I hear are produced by my own footsteps/body” (Agency); “It seems the feeling of my body is less vivid than normal” (Vividness); “The feelings about my body are surprising and unexpected” (Surprise); “It seems like I could really tell where my feet are” (Feet localization).

d) Assessment of changes in emotional state (“questionnaire on emotional feelings”): Emotional valence, dominance, and arousal felt by participants were quantified by using the 9-item graphic scales of the self-assessment manikin questionnaire (Bradley and Lang, 1994).

e) Assessment of changes in gait patterns: Gait biomechanics were taken as an implicit measure of changes in perceived body weight (Tajadura-Jiménez et al., 2015a). The “stance” and the “swing” of the two phases of a gait cycle (i.e., the time between two successive steps made by one foot, Cunado et al., 2003) were analyzed. The stance phase starts with the strike of the heel on the ground and ends when the toes lose contact with the ground. Data from the FSR sensors placed on the sandal insoles were used to quantify the mean exerted force of the heel and toes against the ground and their contact times, as well as the stance and the gait cycle times. The swing phase starts with the foot lifting, first accelerating and then decelerating (midswing) while preparing for the next heel strike and while the other foot is on the ground. The foot accelerates again when the flexor muscles are activated to move the foot forward and downwards (Vaughan et al., 1992). The accelerometers data were used to quantify the foot lifting acceleration (as in Tajadura-Jiménez et al., 2015a).

**Figure 3:**
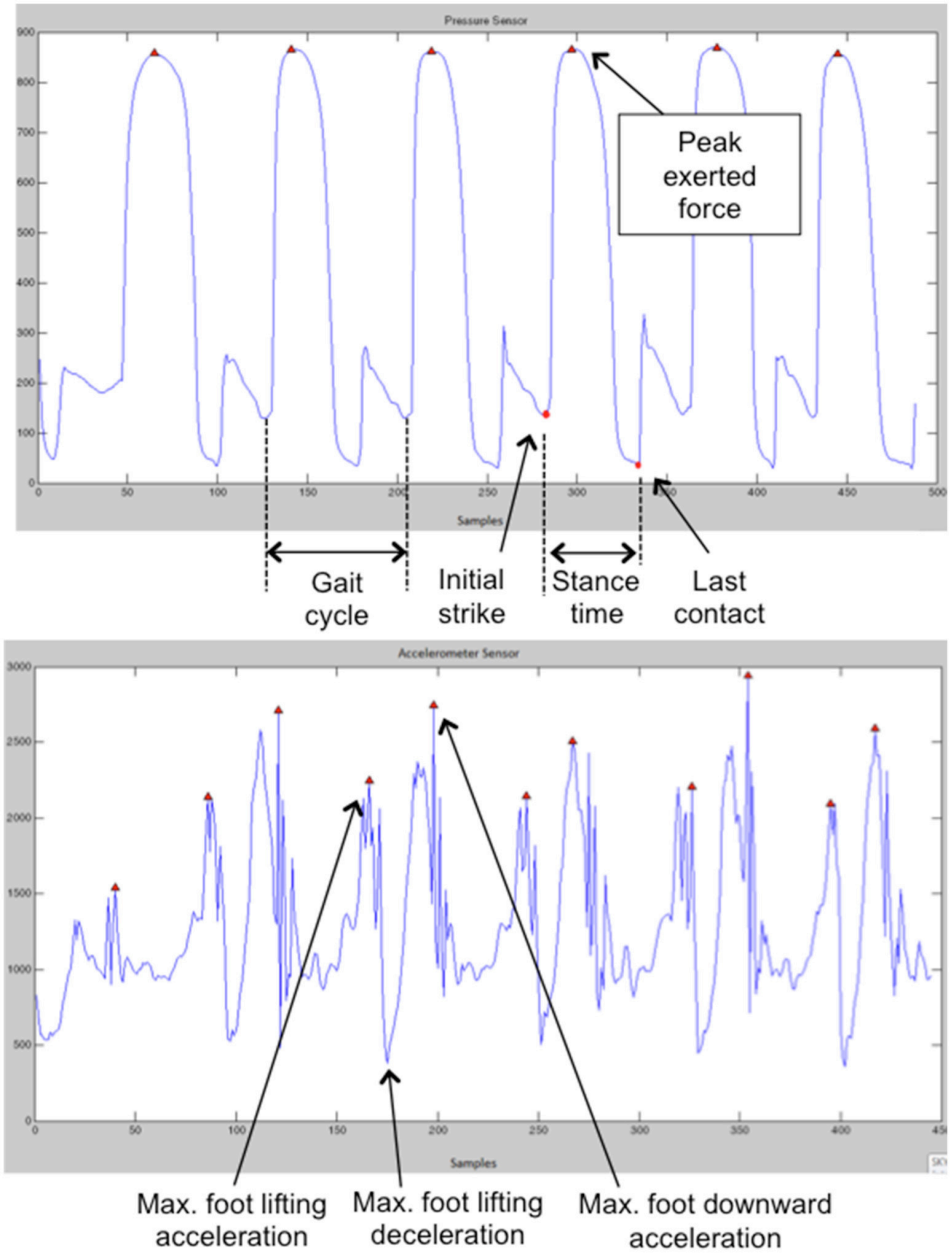
**(Top)** Examples of FSR and **(Bottom)** and accelerometer data. This figure is published in color in the online version.

To extract the gait parameters a specifically developed piece of software was used. Raw sensor data are parsed by this software, which then isolates the accelerometer and FSR readings and creates separate data sets for the left and right foot. FSR data for heel and toe are separated further. As in the paper by Tajadura-Jiménez et al. (2015a), the net acceleration is calculated as the square root of the sum of the squares of the three acceleration axes. The resultant acceleration, FSR of heel and FSR of toe data are low passed filtered to limit the effects of noise (as in Kavanagh and Menz, 2008; Harle et al., 2012). Finally, the first derivative of the resultant acceleration is calculated. For the FSR readings, the software considers that the foot touches the ground when the FSR value exceeds a threshold value. Erroneous detections of the foot leaving the ground are avoided by considering the rate of change of the acceleration readings. Once all steps have been identified within the data sets, the following parameters are extracted for each foot and for each step: mean exerted force of the heel and toes against the ground, stance or contact time (difference between initial strike time and last contact time), gait cycle times and maximum foot lifting acceleration (see Figure 3). For each trial and for each extracted parameter we calculated the average of all steps in the walking phase.

### Procedure

Verbal and written instructions about the tasks were given to participants at the beginning of the session. Next, participants' actual weight and height were recorded as reference. We also asked participants to indicate which was the part of their limb that was more affected (e.g., the knee, the ankle, etc.), and the actual width of this affected body part was also recorded as reference. This body part would be the one referred to during the “aperture” task and the “hands” task. Participants were then asked to complete, in this order, the questionnaire on emotional feelings, the questionnaire on body feelings, the SF-MPQ and the CRPS BPD Scale. Next, participants were equipped with all the sensors and sound-feedback system and were instructed on the tasks for the experiment. They were asked to complete a set of three experimental blocks differing in the sound feedback condition (“Low frequency,” “High frequency,” and “Control”) and presented in a randomized order. In each block, participants were asked to walk on the spot for 60 s, at a self-paced, comfortable speed, while holding the parallel bars on the sides of the treadmill platform. After this 60-s period, participants were asked to complete twice the “aperture” task. In one of the “aperture” trials the lines started together and in the other trial they started 54 cm apart. The order of presentation of these two conditions was randomized, and an average of the two measures was calculated for each of the sound condition (as in Keizer et al., 2013). After the “aperture” task, they completed twice the “avatar” task. The avatar would be proportional to the participant in terms of gender and height, but its initial weight would either be 25% more, or 25% less compared to the participant's actual weight. The order of the two was randomized, and an average of these two weight measures in kilograms was calculated (as in Tajadura-Jiménez et al., 2015a). Finally, after the “avatar” task they completed once the “hands” task, in which participants were asked to close their eyes and used their hands in parallel separated a distance that corresponded to their felt width of their affected body part. The experimenter measured this distance by using a ruler. After providing these body size estimates, participants were removed of the headphones and backpack, and then asked to sit down and to complete in this order, the questionnaire on emotional feelings, the questionnaire on body feelings, the SF-MPQ, and the CRPS BPD scale. Prior to the three experimental blocks, participants performed an initial practice block which was similar to the experimental blocks in terms of tasks and in which they wore the headphones through which they could listen to non-manipulated versions of their footsteps sounds in order to familiarize themselves with the task and the sound feedback.

### Data analyses

We analyzed normal parametrical data (normality tested with Shaphiro–Wilk) with repeated measures analyses of variance (ANOVA), with sound condition (“Control,” “High frequency,” and “Low frequency”) as within-subject factor, except for the gait data for which we conducted for each parameter an ANOVA with 3 × 2 within-subject factors sound condition (“Control,” “High frequency,” and “Low frequency”) and foot (left or right). Significant effects were followed by paired samples two-tailed *t*-tests, with the significance alpha level adjusted for multiple comparisons. We analyzed non-parametrical data with Friedman tests with sound condition (“Control,” “High frequency,” and “Low frequency”) as within-subject factor. Significant effects were followed by Wilcoxon tests, with the significance alpha level adjusted to multiple comparisons. Given the group sizes, we did not use statistical tests for comparison within the four BPD subgroups we identified based on the pre-test body-representation drawings (“Big,” “Small,” “Mixed,” and “Nothing” groups, as described in the Results Section) but we discuss the observed trends for each subgroup as displayed in figures and tables as these trends may provide some insight and inform the design of a larger study conducted in order to establish whether the type of BPD modulates the effect of sound feedback in CRPS.

## Results

### Pre-test values

As previously indicated, pre-test body-representation drawings were produced based on participants verbal descriptions of their body perception when asked to visualize it with eyes closed as part of the CRPS BPD scale. The results of this indicated four types of BPD (see Table 1): “Big” (i.e., limb represented as unusually big; 3 participants), “Small” (i.e., limb represented as unusually small; 1 participant), “Nothing” (i.e., not able to visualize his/her limb; 6 participants), and “Mixed” (i.e., a mixture of two or all the other groups; 2 participants). An example of each BPD group is displayed in Figure 1. The pre-test values for each individual corresponding to BPD scores, actual and estimated body dimensions, reported pain, emotional and bodily feelings are presented in Tables S1–S4. As previously mentioned, an analysis of the above by BPD group is out the scope of this study given the small population; a qualitative analysis, instead, aims to provide some insight into whether the type of BPD modulates the effect of sound feedback in CRPS.

**Figure 4:**
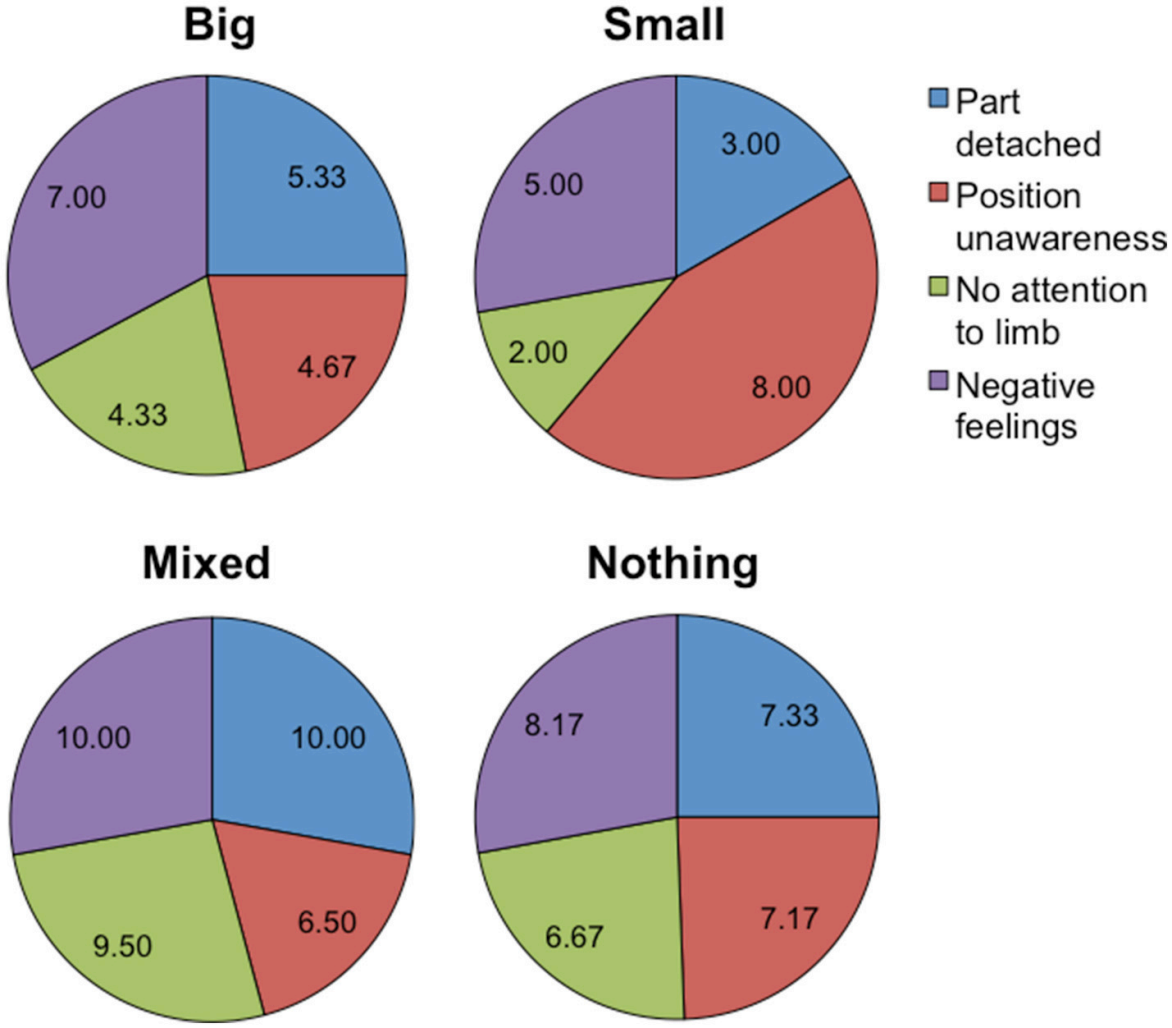
Pie charts summarizing the mean pre-test body perception disturbance scores (*N* = 12). The four charts correspond to the four body disturbance groups: “Big” (*N* = 3), “Mixed” (*N* = 2), “Small” (*N* = 1), “Nothing” (*N* = 6). The pie sectors correspond to the first four 11-level Likert items of the Bath CRPS Body Perception Disturbance questionnaire. For the item “Part detached” the scale ranges from “very much a part” (0) to “completely detached” (11); for the item “Position unawareness” the scale ranges from “very aware” (0) to “completely unaware” (11); for the item “No attention to limb” the scale ranges from “full attention” (0) to “no attention” (11); for the item “Negative feelings” the scale ranges from “strongly positive” (0) to “strongly negative” (11). This figure is published in color in the online version.

Table 2 summarizes the pre-test CRPS total score (CRPS BPD), the ratio between estimated and actual body dimensions, reported pain and emotional feelings, for each BPD group. As it can be seen, CRPS total scores and pain scores were higher in the “Mixed” and “Nothing” groups than in the “Big” and “Small” groups. As shown in Figure 4, in the “Mixed” and “Nothing” groups, BPD scores for feelings of one's body part being detached, not paying attention to limb and negative feelings were higher than in the “Big” and “Small” groups; the feelings of body part position unawareness were higher for the “Small” and the “Nothing” groups than for the “Big” and “Mixed” groups.

**Table 2:** Mean (±SE) CRPS score, ratio between estimated and actual body dimensions, reported pain, and emotional feelings during the pre-test for each participant, according to their BPD group.

**BPD group**	**CRPS Total score**	**Ratio between estimated and actual body dimensions**			**SF-MPQ Pain scores**				**Emotional feelings**		
		**Avatar task**	**Aperture task**	**Hands task**	**Sensory descript**.	**Affective descript**.	**PPI**	**VAS**	**Val**	**Aro**	**Dom**
Big	26.33 (3.84)	0.96 (0.05)	1.58 (0.23)	1.61 (0.24)	9.00 (0.58)	0.00 (0.00)	1.67 (0.33)	5.62 (1.47)	5.33 (0.33)	5.67 (0.33)	3.67 (0.67)
Mixed	41.50 (3.50)	1.22 (0.05)	1.73 (0.1)	1.89 (0.11)	19.50 (0.50)	6.00 (4.00)	3.50 (0.50)	6.83 (0.08)	4.50 (2.50)	5.50 (2.50)	3.50 (1.50)
Small	23.00 (0)	1.15 (0)	2.76 (0)	2.13 (0)	14.00 (0)	3.00 (0)	2.00 (0)	6.25 (0)	7.00 (0)	5.00 (0)	5.00 (0)
Nothing	35.33 (2.76)	0.86 (0.09)	2.05 (0.4)	1.73 (0.31)	15.50 (3.89)	5.17 (1.64)	3.17 (0.40)	6.33 (0.60)	6.00 (1.03)	5.33 (0.56)	4.33 (1.15)

*Estimates of body weight were quantified by the “avatar” task and estimates of the width of the affected body part were quantified by the “aperture” and the “hands” tasks. SF-MPQ scores include sensory and affective descriptors, Present Pain Intensity (PPI) score, VAS pain intensity score. Ratings of emotional feelings include emotional valence (Val), arousal (Aro), and dominance (Dom). The SF-MPQ scores correspond to the sum of the intensity rank values of the words chosen for sensory and affective descriptors. The PPI score ranges from 0 (no pain) to 5 (excruciating). The VAS pain score is a value between 0 and 10 cm, corresponding to a visual analog rating scale. Valence, Arousal, and Dominance ratings refer to the 9-item graphic scales of the self-assessment Manikin questionnaire*.

Comments from the “Mixed” and “Nothing” groups when asked to visualize their body in order to produce the body visualization drawings, include the following (see also comments in Figure 1):

Mixed:

- “my right arm feels shorter, but hand and fingers are fatter; my right hip feels like a football and my right leg seems to end above the knee” (Participant P03)
- “my right arm feels heavy and slightly bigger than the left, and the hand feels twice bigger and longer; my right tight feels heavy and much bigger than normal, with some parts missing; I cannot visualize the right calf but it feels heavy and cold” (Participant P08).

Nothing:

- “I cannot visualize at all the left side of my body, but left arm and left leg feel heavy” (Participant P06)
- “I cannot visualize my left leg from half tight down, it is too painful; I don't know what to think about it, I hate it” (Participant P09)
- “my left tight feels skinny and I can't visualize the leg from above the knee down; the right knee feels ugly and distorted” (Participant P11)
- “I cannot visualize most of my left body (no arm, no leg) but feels heavy” (Participant P02)
- “below the knee the leg is blurry, it feels wooden (“like a pirate leg”); I don't know if it is big or small but it feels cold and wet” (Participant P05)
- “it is difficult to visualize the left arm; it is blurry and feels different than right arm and heavy” (Participant P12).

### Effects of sound condition in BPD

The sections below summarize the effects of sound feedback received during the 1-min walking periods on the alteration of BPD. These effects were quantified in three different ways. First, by assessing the effect of sound on perceived body dimensions, using the avatar, aperture and hands task. Second, by quantifying changes on gait mechanics, as an implicit measure of changes in perceived body weight. And third, by looking at the effect of sound on CRPS descriptors (including drawings), pain and other bodily/emotional feelings that may indicate changes in perceived body parts.

#### Effect of sound condition on perceived body dimensions

**Figure 5:**
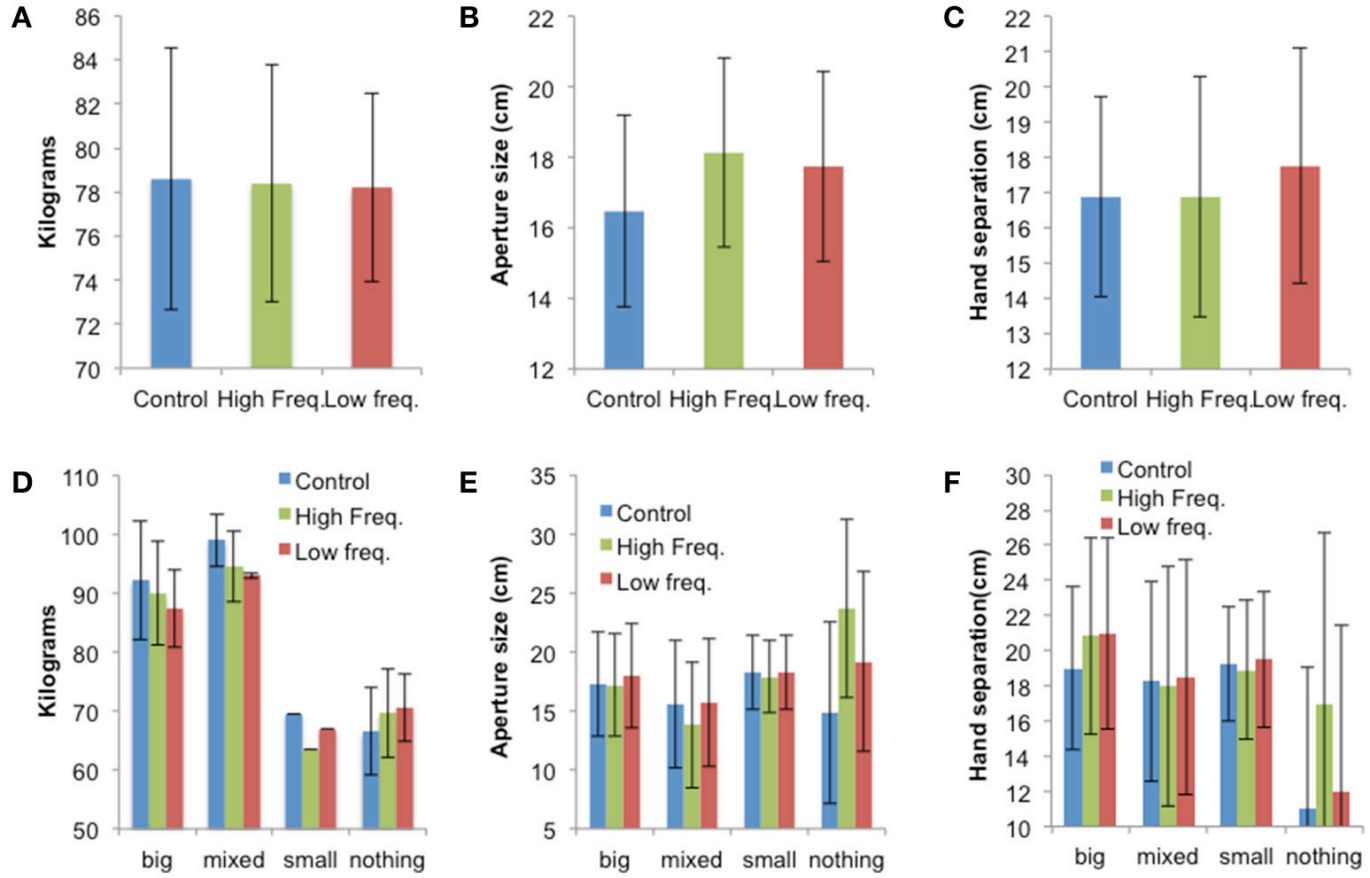
**(A)** Mean perceived body weight (±SE), **(B)** mean aperture size (±SE), and **(C)** mean hand separation (±SE) for all three sound conditions (*N* = 12). Panels **(D–F)** show the mean results (±SE) for each BPD group: “Big” (*N* = 3), “Mixed” (*N* = 2), “Small” (*N* = 1), “Nothing” (*N* = 6). This figure is published in color in the online version.

The mean values ± SE for the “avatar,” “aperture,” and “hands” tasks for all sound conditions (“Control,” “High frequency,” and “Low frequency”) are presented in Figure 5 and individual data are presented in Tables S5–S7. While there were no statistically significant effects of sound on perceived body dimensions, the results shown in Figure 5 suggest that the sound condition affects perceived body dimensions differently according to the BPD group. This is evident on the data from the “aperture” and “hands” tasks. Indeed, Figures 5E,F suggest that sound feedback has larger effect on participants not able to visualize their affected body part (i.e., from the “Nothing” group). These participants represented their body part larger in the sound conditions, especially in the “High frequency” condition compared to the no sound (“Control”) condition.

#### Effect of sound condition on pain

**Figure 6:**
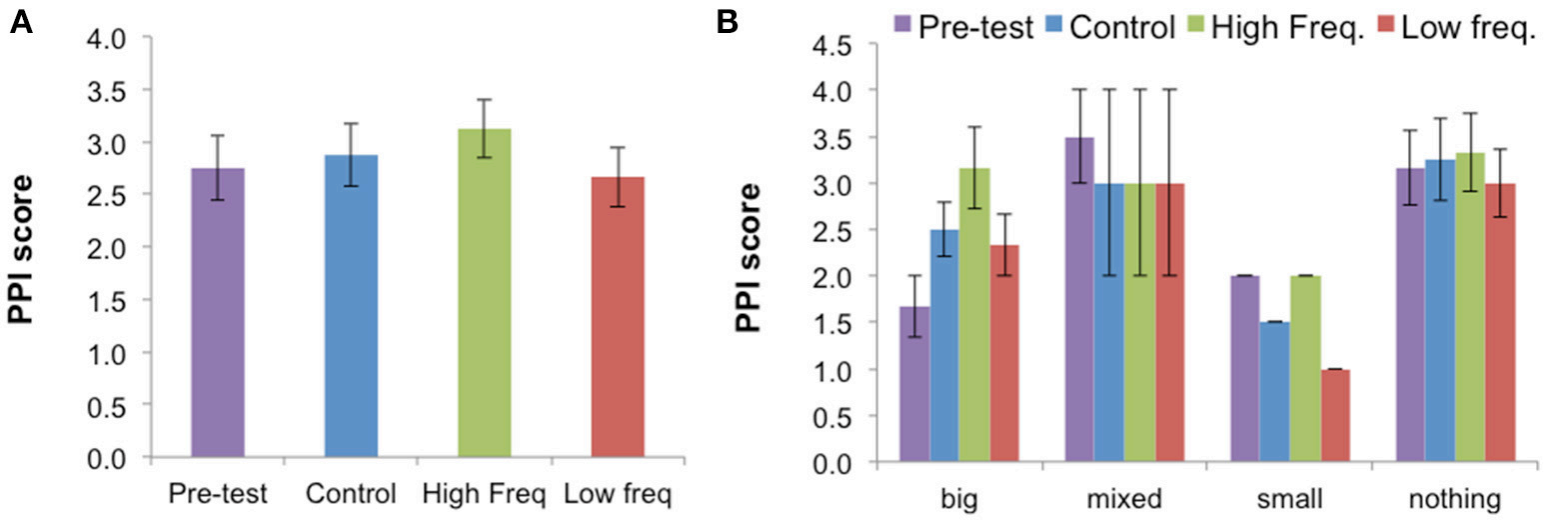
**(A)** Mean Present Pain Intensity (PPI) score (±SE) for all three sound conditions and pre-test condition for all participants (*N* = 12). **(B)** Mean results (±SE) for each BPD group: “Big” (*N* = 3), “Mixed” (*N* = 2), “Small” (*N* = 1), “Nothing” (*N* = 6). The PPI (Present Pain Intensity) index is a pain score ranging from 0 (no pain) to 5 (excruciating). This figure is published in color in the online version.

The mean values ± SE for the McGill Pain PPI index for the pre-test and for all sound conditions (“Control,” “High frequency,” and “Low frequency”) are presented in Figure 6. The mean values ± SE for the other McGill Pain data are presented in Figure S1 and the individual data are presented in Tables S8, S9.

Reviewing the effects of sound on the sensory descriptors, affective descriptors, total descriptors, and on the PPI and visual analog rating scale (VAS) of pain intensity, only the PPI showed a significant effect of sound condition [X^2^(2) = 7.28, *p* = 0.026]. Follow-up Wilcoxon tests showed that the “High frequency” condition elicited higher ratings of pain than the “Low frequency” condition (*Z* = −2.33, *p* = 0.020). The effect of sound on the PPI suggests an effect of sound on the *unpleasantness* dimension of pain quantified by this index, rather than in the *intensity* dimension of pain, which the VAS scale quantifies. Data from each BPD group suggests that the sound condition may affect the pain ratings differently according to the BPD group.

#### Effect of sound condition on CRPS descriptors and other bodily/emotional feelings

**Figure 7:**
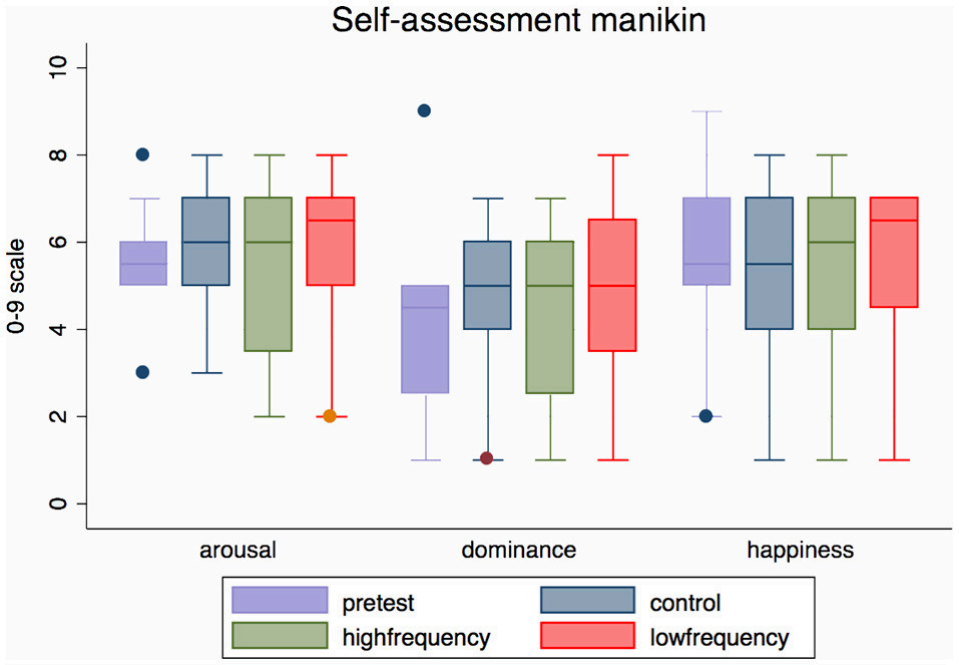
Median (range) emotional arousal, dominance and happiness (valence) for all three sound conditions and pre-test condition for all participants (*N* = 12). Valence, Arousal, and Dominance ratings refer to the 9-item graphic scales of the self-assessment Manikin questionnaire. This figure is published in color in the online version.

Participants reported that sound did have an effect on how the CRPS affected limb felt, and their associated bodily and emotional feelings. Group data (Median and range) are displayed in Tables 3–5 and Figure S2 and the individual data are presented in Tables S10–S12. These did not reach statistical significance except for the dominance scale [*X*^2^(2) = 6.70, *p* = 0.035]. People reported feeling more dominant in the Low frequency condition than in the High frequency condition (*Z* = −2.33, *p* = 0.020), with the rating for the Control condition falling in between the other two ratings (see Figure 7 and Table 3).

**Table 3:** Emotional valence (Val), Arousal (Aro), and Dominance (Dom) for all three sound conditions according to BPD group.

**BPD group**	**Control condition**			**High frequency condition**			**Low frequency condition**		
	**Val**	**Aro**	**Dom**	**Val**	**Aro**	**Dom**	**Val**	**Aro**	**Dom**
“Big”	5 (3–8)	6 (5–7)	5 (3–7)	6 (3–7)	7 (3–7)	6 (2–6)	7 (3–7)	7 (6–7)	7 (3–7)
“Mixed”	3.5 (1–6)	5.5 (3–8)	3 (1–5)	4 (1–7)	5 (2–8)	3 (1–5)	4 (1–7)	5 (2–8)	3 (1–5)
“Small”	7	7	6	7	6	4	7	7	6
“Nothing”	5.5 (2–7)	6 (4–7)	5.5 (2–7)	6 (2–8)	5.5 (3–7)	5 (2–7)	6 (2–7)	6 (4–7)	5 (2–8)
All participants	5.5 (1–8)	6 (3–8)	5 (1–7)	6 (1–8)	6 (2–8)	5 (1–7)	6.5 (1–7)	6.5 (2–8)	5 (1–8)

*The values correspond to 9-level Likert items of the self-assessment Manikin questionnaire (Median value and range are indicated)*.

**Table 4:** Bath CRPS Body perception Disturbance questionnaire data for all three sound conditions according to BPD group.

**Condition**	**BPD group**	**Part detached**	**Position unawareness**	**No attention to limb**	**Negative feelings**	**Change size**	**Change temperature**	**Change pressure**	**Change weight**
Control	“Big”	7 (4–8)	7 (5–8)	5 (1–7)	8 (3–8)	3	3	3	3
	“Mixed”	10 (10–10)	6.5 (3–10)	10 (10–10)	10 (10–10)	2	2	2	2
	“Small”	3	3	4	5	1	1	1	1
	“Nothing”	8.5 (5–10)	8.5 (5–9)	8 (4–10)	7.5 (6–10)	5	6	4	6
	All participants	7 (2–10)	7.5 (3–10)	7 (1–10)	8 (3–10)	11	12	10	12
High frequency	“Big”	8 (3–9)	8 (5–9)	7 (5–7)	7 (3–8)	3	3	3	3
	“Mixed”	9.5 (9–10)	6.5 (3–10)	10 (10–10)	10 (10–10)	2	2	2	2
	“Small”	4	0	2	5	1	1	1	1
	“Nothing”	5 (3–10)	4.5 (3–10)	4 (3–10)	7.5 (4–10)	5	6	6	6
	All participants	7 (3–10)	5 (0–10)	6 (2–10)	7.5 (3–10)	11	12	11	12
Low frequency	“Big”	7 (2–7)	8 (5–8)	5 (1–7)	7 (5–8)	2	3	3	3
	“Mixed”	9.5 (9–10)	6.5 (3–10)	10 (10–10)	10 (10–10)	2	2	2	2
	“Small”	7	7	7	3	0	1	0	1
	“Nothing”	7 (3–10)	6.5 (3–9)	6 (3–10)	8.5 (5–10)	5	5	5	6
	All participants	8 (3–10)	7.5 (3–10)	7 (1–10)	8 (3–10)	9	11	10	12

*The values correspond to 11-level Likert items for the first four items (Median value and range are indicated) and frequency data for the other four items. For the item “Part detached” the scale ranges from “very much a part” (0) to “completely detached” (11); for the item “Position unawareness” the scale ranges from “very aware” (0) to “completely unaware” (11); for the item “No attention to limb” the scale ranges from “full attention” (0) to “no attention” (11); for the item “Negative feelings” the scale ranges from “strongly positive” (0) to “strongly negative” (11)*.

**Table 5:** Body feelings questionnaire data for all three sound conditions according to BPD group.

**Condition**	**BPD group**	**Speed**	**Weight**	**Strength**	**Extended**	**Agency**	**Vividness**	**Surprise**	**Feet localization**
Control	“Big”	3 (2–4)	6 (2–6)	4 (3–5)	4 (2–6)	4 (4–6)	5 (3–5)	4 (4–5)	4 (2–6)
	“Mixed”	2.5 (1–4)	6 (5–7)	2.5 (1–4)	3.5 (3–4)	2.5 (1–4)	2 (1–3)	5.5 (4–7)	2 (1–3)
	“Small”	4	3	4	5	2	2	6	5
	“Nothing”	2 (1–6)	6 (5–6)	2 (1–5)	2 (1–5)	3 (1–6)	4 (1–6)	4.5 (2–7)	3.5 (1–6)
	All participants	2.5 (1–6)	6 (2–7)	3 (1–5)	3.5 (1–6)	3.5 (1–6)	3.5 (1–6)	4.5 (2–7)	3.5 (1–6)
High frequency	“Big”	4 (3–6)	3 (3–7)	4 (1–5)	5 (1–6)	3 (1–6)	2 (2–4)	5 (2–6)	5 (1–6)
	“Mixed”	2.5 (1–4)	6 (5–7)	2 (1–3)	3.5 (3–4)	4 (1–7)	2 (1–3)	5.5 (4–7)	3 (3–3)
	“Small”	3	5	2	2	6	4	2	7
	“Nothing”	3 (1–5)	5 (3–7)	2 (2–4)	2.5 (2–5)	5 (1–6)	5 (2–6)	5 (2–6)	5 (1–5)
	All participants	3 (1–6)	5 (3–7)	2 (1–5)	3 (1–6)	5 (1–7)	3.5 (1–6)	5 (2–7)	5 (1–7)
Low frequency	“Big”	5 (2–5)	4 (2–6)	4 (2–5)	6 (2–6)	6 (2–6)	6 (3–6)	5 (2–6)	5 (2–6)
	“Mixed”	2.5 (1–4)	5.5 (4–7)	2.5 (1–4)	3 (3–3)	7 (7–7)	2.5 (1–4)	5.5 (4–7)	4.5 (3–6)
	“Small”	5	3	4	6	6	2	5	6
	“Nothing”	2.5 (1–6)	6 (5–6)	2.5 (2–4)	2.5 (1–5)	5 (1–6)	3.5 (2–6)	5 (3–6)	3.5 (2–5)
	All participants	3 (1–6)	6 (2–7)	3.5 (1–5)	3 (1–6)	5.5 (1–7)	3.5 (1–6)	5 (2–7)	4.5 (2–6)

*The values correspond to 7-level Likert items (Median value and range are indicated). For the item “Speed” the scale ranges from “slow” (1) to “quick” (7); for the item “Weight” the scale ranges from “light” (1) to “heavy” (7); for the item “Strength” the scale ranges from “weak” (1) to “strong” (7); for the item “Straight” the scale ranges from “crouched, stoop” (1) to “elongated, extended” (7). For the remaining items (“Agency,” “Vividness,” “Surprise,” “Feet localization”), the scale indicates the level of agreement with the statement, ranging from “I strongly disagree” (1) to “I strongly agree” (7)*.

Other reported effects of sound included how detached their limb felt, limb position awareness, attention to the affected limb and negative feelings toward the limb. Some quotes of participants from the “Nothing” group, for which these effects of sound seemed more evident, are given below:

- “below the knee the leg is less blurry than before” (Participant P05—“High frequency” condition); “below the knee the leg is still blurry, feels very thin, but more like a limb than wood.” (Participant P05—“Low frequency” condition);
- “I can now slightly vaguely visualize both of my hips, but nothing else on the left side” (Participant P06—both sound conditions);
- “I have a sense of the left side of the body (arm and leg), even if I don't see them (they are not solid); I can clearly see the left hip.” (Participant P02—“High frequency” condition); “I cannot visualize the left arm and leg, but I can visualize the left hip, which usually I can't” (Participant P02—“Low frequency” condition).

It should be noted that the increase in attention to limb and restoring of body representations sometimes came accompanied by an increase in negative sensations, as in the case of one of the participants from the “Nothing” group, who said: “*I can feel my left arm big all the way down, but I feel more pressure in it (blood pumping)”* (Participant P12—“High frequency” condition); “*I can feel my left arm big all the way down, but the pumping in the hand has increased; I feel more pain and more aware of my arm—If I don't do anything, I feel nothing, I don't see it, I try to forget about it. But you asked me to focus on it and now I visualize it—it feels bigger and painful”* (Participant P12—“Low frequency” condition). Another participant from the “Big” group, however, reported less pain in the sound conditions than in the control condition: “*My knee feels a little uncomfortable behind, touching, a little painful”* (Participant P04—“Control” condition); “*My knee feels a little uncomfortable behind, but less than before”* (Participant P04—“High frequency” condition); “*My knee feels a little uncomfortable behind, but not really. It doesn't hurt”* (Participant P04—“Low frequency” condition).

Although, not significant, for most descriptors of bodily feelings we observed differences between the median scores for the sound conditions and the control condition. In particular, people reported feeling **faster** in both sound conditions than in the “Control” condition, **less heavy** in the “High frequency” condition than in the other conditions and **stronger** in the “Low frequency” condition than in the other conditions. They also felt **more able to localize their feet**, **more surprised about their bodily feelings**, and more **agent of the sounds** in both sound conditions than in the “Control” condition. There were observable differences between groups for most descriptors, as for instance, the effect of the “Low frequency” condition in feelings of strength was more obvious in the “Nothing” group.

We found a clear beneficial effect of one or both sound conditions compared to the control condition for seven out of the twelve participants (one participant from the “Big” group, all three participants comprising the “Small” and the “Mixed” groups, and three participants from the “Nothing” group). For three participants in the “Nothing” group we observed an improvement of the BPD for one or both of the sound conditions as well as the control condition compared to the pre-test, BPD. For two participants in the “Big” group we found that either both sound conditions worsened the participant's body-representation in terms of exacerbating the disturbance, or one of the conditions was worse and the other did not have an effect.

#### Effect of sound condition on gait

**Figure 8:**
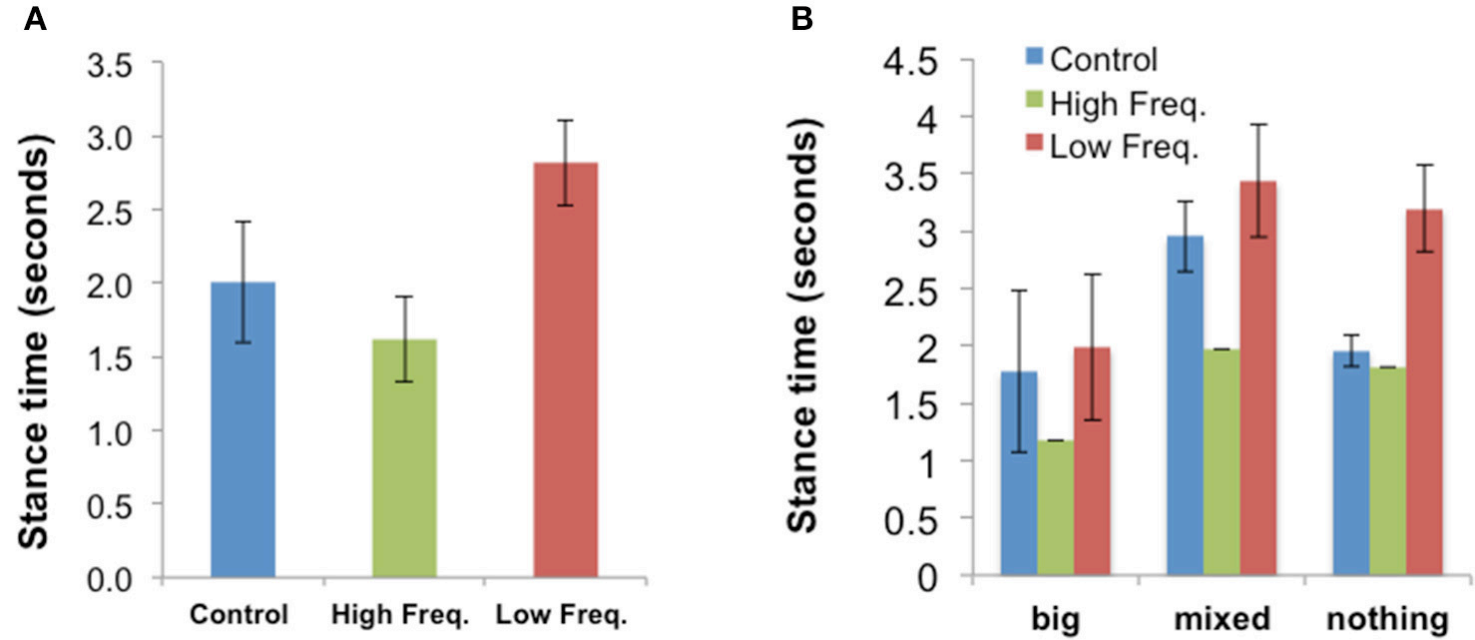
**(A)** Mean stance time (±SE) for all three sound conditions (*N* = 9). **(B)** Shows the mean results for each body disturbance group: “Big” (*N* = 3), “Mixed” (*N* = 1), “Nothing” (*N* = 5). This figure is published in color in the online version.

Gait data for three participants were lost. For the remaining 9 participants, gait parameters were extracted as described in Section Methods. Reviewing the effects of sound on all the gait parameters, the **stance time** showed a significant effect of sound condition [*F*_(2, 16)_ = 3.91, *p* = 0.041]. Participants spent more time with their foot in contact with the ground in the Low frequency condition than in the High Frequency condition [*t*_(8)_ = 3.89, *p* = 0.005], with the average contact time for the Control condition falling in between the time for the other two conditions. A similar related effect of sound condition was found for the **toe contact time** [*F*_(2, 16)_ = 4.62, *p* = 0.026]: People spent more time with their toe in contact with the ground in the Low frequency condition than in the High Frequency condition [*t*_(8)_ = 3.84, *p* = 0.005]. The mean stance times ± SE for all sound conditions (“Control,” “High frequency,” and “Low frequency”) are presented in Figure 8. The mean values ± SE for other gait parameters are presented in Figures S3–S6.

## Discussion

Previous research has shown that sound can affect body perception in healthy controls (Tajadura-Jiménez et al., 2012, 2015a,b, 2016). This proof-of-principle pilot study has suggested that sound can alter body perception in patients with CRPS and chronic pain with body perception disturbances (BPD). However, while in healthy controls the effect of high or low frequency alterations of own walking sounds on body perception is predictable (i.e., the body feels lighter in the high frequency condition than in the low frequency condition; Tajadura-Jiménez et al., 2015a), this relationship is not consistent in CRPS. The patients with CRPS in this study all had BPD of varying types and therefore it is not surprising that the effect of sound would not be as predictable. The data suggests that the type of BPD may influence the effect of sound feedback on body perception. Sound feedback seems to have larger effect on perceived body dimensions and on CRPS descriptors in participants not able to visualize their affected body part (i.e., from the “Nothing” group). A much larger study would be required to establish whether the type of BPD modulates the effect of the frequency of sound feedback in CRPS, or whether in the context of chronic pain and BPD the frequency has any importance or it is more an effect of sound per-se and/or an interaction with attention and distractive factors.

In the current study the effects of sound feedback received during the 1-min walking periods on the alteration of BPD were quantified in three different ways: by assessing the effect of sound on perceived body dimensions; by quantifying the effects on the related gain mechanics; and by looking at the effect of sound on CRPS descriptors, pain, and other bodily/emotional feelings, which may indicate changes in perceived body parts. Our data suggest that sound feedback can affect the perceived size of the CRPS affected limb, and the observed trends within BPD subgroups suggest that this may differ according to the type of BPD. Effects on perceived body dimensions were more evident on the data assessing specifically the perceived size of the affected limb (i.e., “aperture” and “hands” tasks), than in the data assessing the perceived overall body size (i.e., data collected with the avatar tool). It is important to take into account that this avatar tool we used, which we adopted from our previous study with healthy population (Tajadura-Jiménez et al., 2015a), did not allow modifying the size of the individual limbs of the avatar. An avatar tool allowing modifications of all body parts has been used previously to explore BPD in CRPS (Turton et al., 2013). Patients found this an acceptable tool for communicating their BPD, and described a positive impact being able to see an image they had previously only imagined. Peltz and colleagues used schematic drawings to explore hand size perception in upper limb of people with CRPS, and found a tendency to overestimation which correlated with disease duration, neglect score, and increase of two-point-discrimination-thresholds (Peltz et al., 2011). Other work has revealed that more extensive BPD is associated with worse tactile acuity, and correlates positively with pain (Lewis and Schweinhardt, 2012). In our study, the schematic drawings of participants' body visualizations revealed both tendencies to overestimation and underestimation with some participants not able to visualize parts of their body (those in the “Nothing” group or “Mixed” groups). The qualitative data suggest that sound may cause these body parts to remerge.

We demonstrated that sound feedback can affect the pain experienced in CRPS, and that this is bidirectional (i.e., pain may increase or decrease with sound) and may vary according to the type of BPD. It has been previously demonstrated that ambiguous visual stimuli can enhance pain in CRPS (Hall et al., 2011; Cohen et al., 2012). In our study, the qualitative descriptors from one of the participants in the “Nothing” group suggested that when the sound feedback enhanced the awareness of the affected limb, it resulted in increased pain. Neglect-like phenomena are recognized in CRPS (Kolb et al., 2012), and this participant described using neglect-like strategies to cope. Therefore, the type of BPD may be an important factor in determining how sound feedback may affect CRPS pain. We also found that sound feedback affected CRPS descriptors and other bodily feelings and emotions including feelings of emotional dominance, limb detachment, position awareness, attention and negative feelings toward the limb. Future work would need to carefully phenotype patients and explore their particular BPD and bodily feelings and emotions in order to better understand how to utilize sound feedback optimally.

We also demonstrated an effect of sound feedback on gait. Time of foot contact with the ground increased in the low frequency condition compared to the high frequency condition. This is consistent with previous work in healthy controls where in the high frequency condition, participants experience their body as lighter, the time of foot contact with the ground reduces and the foot lifting acceleration increases in a way consistent with actually having a lighter body (Tajadura-Jiménez et al., 2015a). This may have relevance to rehabilitation, particularly where lower limb CRPS patients perceive the limbs as heavy and weak, which may contribute to the gait impairment that is often observed in CRPS population (Galer et al., 1999). Visual manipulation is established in CRPS treatment in mirror visual feedback therapy (Méndez-Rebolledo et al., 2016), and in therapies using prisms (Moseley et al., 2013). There is potential to combine manipulation of auditory and visual stimuli in the treatment of CRPS and future work would be needed to discover if this is practical, and offers the potential for a synergistic effect.

The possibility of using sensory feedback to “retrain” the brain of people with CRPS might offer a new treatment approach. Alterations in the somatosensory cortex are thought to be behind the anomalous bodily experiences of people with CRPS (Flor et al., 1995, 1997; Maihöfner et al., 2003, 2004; McCabe et al., 2003; Pleger et al., 2005; Marinus et al., 2011) and previous studies using sensory feedback to manipulate people's body representations have linked their results to recalibration of somatosensory receptive fields (RF) in the somatosensory cortex (Taylor-Clarke et al., 2004; de Vignemont et al., 2005; Haggard et al., 2007; Cardinali et al., 2009, 2012; Cardini et al., 2011, 2012, 2013; Tajadura-Jiménez et al., 2012, 2016; Canzoneri et al., 2013a,b; Miller et al., 2014; Cardini and Longo, 2016). We suggest that the observed changes in body-representation in the current study may also indicate reorganization within the somatosensory cortex The observed changes in kinematics of gait may also support this suggestion, if it is considered that the control of body movements relies on somatosensory representations of body dimensions (Holmes and Spence, 2004; Maravita and Iriki, 2004; Cardinali et al., 2009; Tajadura-Jiménez et al., 2016). Consistent with the theories of “forward internal models” of motor-to-sensory transformations (Wolpert and Ghahramani, 2000), body-representations are used among other inputs when planning actions and predicting the sensory feedback (e.g., the sound of one's footsteps) that should be received from such actions. When the sensory feedback received from one's actions does not match these predictions, an update of the internal somatosensory body model may occur. It has been suggested that the observed gait changes may result from an attempt to reduce the sensory discrepancies introduced by the sound feedback, and that these gait changes may contribute to maintain the bodily illusion induced by the sound (Tajadura-Jiménez et al., 2015a). It is possible that changes in body perception, emotion and gait, may reinforce each other during the period of exposure to the stimulation.

This is a proof-of-principle pilot study and thus there are limitations in the design and generalization of findings. The most significant limitation is the number of participants; this is a consistent difficulty encountered in clinical studies of CRPS (O'Connell et al., 2013) due to the relatively rare nature of the condition and difficulties in recruitment. This could be addressed in future studies and by multicenter collaboration. Our study had a predominance of lower limb affected CRPS patients, but data on our upper limb affected CRPS patients suggest that the effects of manipulating footstep sounds may extend to other body parts apart from lower limbs. Further work should aim to balance the distribution of affected limbs, and establish whether the limb/s affected has any relevance upon the effect and utility of sound feedback. The participants in our study also had a wide range of disease duration and future work with larger numbers should characterize whether this also a significant factor. Our work has demonstrated the possible importance of the type of BPD and further work should aim to explore the CRPS phenotype in detail including the BPD and associated emotions and bodily feelings together with other potentially linked aspects such as tactile discrimination (Peltz et al., 2011; Lewis and Schweinhardt, 2012) and neglect-like phenomena (Kolb et al., 2012). Our research has demonstrated that sound feedback can affect BPD and pain, and may potentially inform the design of currently available sensorimotor based therapy combining visual, tactile and motor strategies; this should be explored in clinical studies with CRPS and other patients with chronic pain and BPD such as fibromyalgia and phantom limb phenomena in amputees.

## Conclusion

Our results suggest that sound feedback may be used to alter body perception and its related emotional state and gait in those with CRPS. They suggest that sound feedback may affect the perceived size of the CRPS affected limb and the pain experienced, but that the effects may differ according to the type of body perception disturbance. Further, there are indications that sound feedback affected CRPS descriptors and other bodily feelings and emotions including feelings of emotional dominance, limb detachment, position awareness, attention, and negative feelings toward the limb. Gait varied with sound feedback, affecting the foot contact time with the ground in a way consistent with experienced changes in body weight. These findings may inform the experiment protocol for larger studies and have potential application for regenerating altered body-representation and its related bodily feelings in a clinical setting for patients with chronic pain and body perception disturbances.

## Author contributions

All authors contributed to the conception and design of the work, interpretation of data and revision of the drafts of the work. AT acquired and analyzed the data, and drafted the work. All authors agreed to be accountable for all aspects of the work in ensuring that questions related to the accuracy or integrity of any part of the work are appropriately investigated and resolved, and approved this final version of the manuscript.

### Conflict of interest statement

The authors declare that the research was conducted in the absence of any commercial or financial relationships that could be construed as a potential conflict of interest.
