# Supplementary material for: Bodily Sensory Inputs and Anomalous Bodily Experiences in Complex Regional Pain Syndrome: Evaluation of the Potential Effects of Sound Feedback
Source: Front Hum Neurosci. 2017 Jul 27;11:379. doi: 10.3389/fnhum.2017.00379 (PMC5529353; doi:10.3389/fnhum.2017.00379)
Supplement: Supplementary file 1 [file Table1.DOCX]

**Table S1. Pre-test values for Bath CRPS Body Perception Disturbance questionnaire data for each participant according to the body disturbance group.** The values correspond to 11-level Likert items from 0 to 10 for the first four items and frequency data for the other four items. For the item “Part detached” the scale ranges from “very much a part” (0) to “completely detached” (11); for the item “Position unawareness” the scale ranges from “very aware” (0) to “completely unaware” (11); for the item “No attention to limb” the scale ranges from “full attention” (0) to “no attention” (11); for the item “Negative feelings” the scale ranges from “strongly positive” (0) to “strongly negative” (11).

| **Distortion group** | **P Id** | **Part detached** | **Position unawareness** | **No attention to limb** | **Negative feelings** | **Change Size** | **Change Temperature** | **Change Pressure** | **Change Weight** |
| --- | --- | --- | --- | --- | --- | --- | --- | --- | --- |
| ‘Big’ | P04 | 5 | 3 | 5 | 5 | 0 | 1 | 1 | 1 |
|  | P10 | 4 | 4 | 1 | 8 | 1 | 1 | 1 | 1 |
|  | P07 | 7 | 7 | 7 | 8 | 1 | 1 | 1 | 1 |
| ‘Mixed’ | P03 | 10 | 10 | 9 | 10 | 1 | 1 | 1 | 1 |
|  | P08 | 10 | 3 | 10 | 10 | 1 | 1 | 1 | 1 |
| ‘Small’ | P01 | 3 | 8 | 2 | 5 | 1 | 1 | 1 | 1 |
| ‘Nothing’ | P05 | 7 | 7 | 5 | 8 | 1 | 1 | 1 | 1 |
|  | P12 | 8 | 8 | 10 | 10 | 1 | 1 | 1 | 1 |
|  | P09 | 8 | 8 | 2 | 9 | 1 | 1 | 1 | 1 |
|  | P11 | 10 | 9 | 10 | 10 | 1 | 1 | 1 | 1 |
|  | P06 | 4 | 3 | 8 | 6 | 1 | 1 | 1 | 1 |
|  | P02 | 7 | 8 | 5 | 6 | 1 | 1 | 1 | 1 |
