# Supplementary material for: Bodily Sensory Inputs and Anomalous Bodily Experiences in Complex Regional Pain Syndrome: Evaluation of the Potential Effects of Sound Feedback
Source: Front Hum Neurosci. 2017 Jul 27;11:379. doi: 10.3389/fnhum.2017.00379 (PMC5529353; doi:10.3389/fnhum.2017.00379)
Supplement: Supplementary file 2 [file Table2.DOCX]

**Table S2. Pre-test values for actual and estimated body dimensions, and their ratios, for each participant, according to their body distortion group.** The unit for body weight is kilograms and for body part is centimeters. Estimates of body weight were quantified by the ‘avatar’ task and estimates of the width of the affected body part were quantified by the ‘aperture’ and the ‘hands’ tasks.

|  |  | **Actual body dimensions** | | **Estimated body dimensions** | | | Ratio between estimated and actual dimensions | | |
| --- | --- | --- | --- | --- | --- | --- | --- | --- | --- |
| **Distortion group** | **P Id** | **Body weight** | **Width affected part** | **Avatar task** | **Aperture task** | **Hands task** | **Avatar task** | **Aperture task** | **Hands task** |
| ‘Big’ | P04 | 118.50 | 11.50 | 108.50 | 15.32 | 15.5 | 0.92 | 1.33 | 1.35 |
|  | P10 | 60.00 | 11.00 | 54.00 | 22.39 | 23 | 0.90 | 2.04 | 2.09 |
|  | P07 | 74.10 | 7.50 | 78.50 | 10.28 | 10.5 | 1.06 | 1.37 | 1.40 |
| ‘Mixed’ | P03 | 89.30 | 16.00 | 85.50 | 18.72 | 19 | 0.96 | 1.17 | 1.19 |
|  | P08 | 65.80 | 6.00 | 97.50 | 13.75 | 15.5 | 1.48 | 2.29 | 2.58 |
| ‘Small’ | P01 | 65.20 | 8.00 | 75.00 | 22.07 | 17 | 1.15 | 2.76 | 2.13 |
| ‘Nothing’ | P05 | 80.80 | 7.00 | 75.50 | 4.48 | 4.5 | 0.93 | 0.64 | 0.64 |
|  | P12 | 44.30 | 6.50 | 35.50 | 20.02 | missed | 0.80 | 3.08 | missed |
|  | P09 | 101.90 | 12.00 | 110.50 | 30.38 | 32.5 | 1.08 | 2.53 | 2.71 |
|  | P11 | 62.20 | 10.50 | 55.50 | 14.68 | 16 | 0.89 | 1.40 | 1.52 |
|  | P06 | 71.10 | 10.50 | 33.50 | 16.73 | 18.5 | 0.47 | 1.59 | 1.76 |
|  | P02 | 67.00 | 9.00 | 67.00 | 27.46 | 18 | 1.00 | 3.05 | 2.00 |
