# Supplementary material for: Bodily Sensory Inputs and Anomalous Bodily Experiences in Complex Regional Pain Syndrome: Evaluation of the Potential Effects of Sound Feedback
Source: Front Hum Neurosci. 2017 Jul 27;11:379. doi: 10.3389/fnhum.2017.00379 (PMC5529353; doi:10.3389/fnhum.2017.00379)
Supplement: Supplementary file 3 [file Table3.DOCX]

**Table S3. Pre-test values for reported pain and emotional feelings, for each participant, according to their body disturbance group.** Mean SF-MPQ score, PPI score, VAS pain score, emotional valence (Val), arousal (Aro) and dominance (Dom). The SF-MPQ scores correspond to the sum of the intensity rank values of the words chosen for sensory, affective and total descriptors. The PPI (Present Pain Intensity) index is a pain score ranging from 0 (no pain) to 5 (excruciating). The VAS pain score is a value between 0 and 10 cm, corresponding to a visual analogue rating scale. Valence, Arousal and Dominance ratings refer to the 9-item graphic scales of the Self-assessment Manikin questionnaire.

|  |  | **SF-MPQ scores** | | | **Pain levels** | | **Emotional feelings** | | |
| --- | --- | --- | --- | --- | --- | --- | --- | --- | --- |
| **Distortion group** | **P Id** | **Sensory** | **Affective** | **Total** | **PPI** | **VAS** | **Val** | **Aro** | **Dom** |
| ‘Big’ | P04 | 9 | 0 | 9 | 2 | 6.50 | 6 | 5 | 5 |
|  | P10 | 8 | 0 | 8 | 1 | 2.75 | 5 | 6 | 3 |
|  | P07 | 10 | 0 | 10 | 2 | 7.60 | 5 | 6 | 3 |
| ‘Mixed’ | P03 | 19 | 2 | 21 | 3 | 6.75 | 7 | 3 | 5 |
|  | P08 | 20 | 10 | 30 | 4 | 6.90 | 2 | 8 | 2 |
| ‘Small’ | P01 | 14 | 3 | 17 | 2 | 6.25 | 7 | 5 | 5 |
| ‘Nothing’ | P05 | 14 | 2 | 16 | 3 | 6.00 | 5 | 5 | 2 |
|  | P12 | 13 | 8 | 21 | 4 | 6.35 | 5 | 5 | 5 |
|  | P09 | 28 | 8 | 36 | 4 | 6.75 | 9 | 7 | 9 |
|  | P11 | 24 | 10 | 34 | 2 | 8.45 | 2 | 3 | 1 |
|  | P06 | 1 | 0 | 1 | 2 | 3.90 | 8 | 6 | 5 |
|  | P02 | 13 | 3 | 16 | 4 | 6.50 | 7 | 6 | 4 |
