# Supplementary material for: Bodily Sensory Inputs and Anomalous Bodily Experiences in Complex Regional Pain Syndrome: Evaluation of the Potential Effects of Sound Feedback
Source: Front Hum Neurosci. 2017 Jul 27;11:379. doi: 10.3389/fnhum.2017.00379 (PMC5529353; doi:10.3389/fnhum.2017.00379)
Supplement: Supplementary file 5 [file Table5.DOCX]

**Table S5. Results from the ‘aperture’ task (in cm) for all conditions and for each participant according to the body distortion group.**

| **Distortion group** | **Participant id** | **Pre-test** | **Control condition** | **High frequency condition** | **Low frequency condition** |
| --- | --- | --- | --- | --- | --- |
| ‘Big’ | P04 | 15.32 | 12.23 | 11.83 | 12.72 |
|  | P10 | 22.39 | 22.25 | 24.32 | 26.41 |
|  | P07 | 10.28 | 17.26 | 15.29 | 14.71 |
| ‘Mixed’ | P03 | 18.72 | 16.93 | 13.5 | 13.73 |
|  | P08 | 13.75 | 14.27 | 14.15 | 17.63 |
| ‘Small’ | P01 | 22.07 | 14.81 | 23.65 | 19.13 |
| ‘Nothing’ | P05 | 4.47 | 5.45 | 5.74 | 5.78 |
|  | P12 | 20.02 | 25.69 | 21.49 | 21.50 |
|  | P09 | 30.38 | 30.94 | 28.28 | 31.04 |
|  | P11 | 14.68 | 17.38 | 15.25 | 14.68 |
|  | P06 | 16.73 | 17.76 | 25.33 | 16.46 |
|  | P02 | 27.46 | 12.12 | 11.28 | 19.88 |
