# Supplementary material for: Bodily Sensory Inputs and Anomalous Bodily Experiences in Complex Regional Pain Syndrome: Evaluation of the Potential Effects of Sound Feedback
Source: Front Hum Neurosci. 2017 Jul 27;11:379. doi: 10.3389/fnhum.2017.00379 (PMC5529353; doi:10.3389/fnhum.2017.00379)
Supplement: Supplementary file 6 [file Table6.DOCX]

**Table S6. Results from the ‘hands’ task (in cm) for all conditions and for each participant according to the body distortion group.**

| **Distortion group** | **Participant id** | **Pre-test** | **Control condition** | **High frequency condition** | **Low frequency condition** |
| --- | --- | --- | --- | --- | --- |
| ‘Big’ | P04 | 15.5 | 15.50 | 12.00 | 15.00 |
|  | P10 | 23 | 26.00 | 27.50 | 28.00 |
|  | P07 | 10.5 | 15.50 | 23.00 | 20.00 |
| ‘Mixed’ | P03 | 19 | 16.00 | 13.00 | 14.50 |
|  | P08 | 15.5 | 20.50 | 23.00 | 22.50 |
| ‘Small’ | P01 | 17 | 11.00 | 17.00 | 12.00 |
| ‘Nothing’ | P05 | 4.5 | 9.00 | 9.00 | 7.50 |
|  | P12 | not recorded | 12.50 | 14.00 | 18.00 |
|  | P09 | 32.5 | 35.00 | 37.00 | 40.00 |
|  | P11 | 16 | 23.50 | 22.00 | 17.50 |
|  | P06 | 18.5 | 20.00 | 22.50 | 19.50 |
|  | P02 | 18 | 15.50 | 9.00 | 14.50 |
