# Supplementary material for: Bodily Sensory Inputs and Anomalous Bodily Experiences in Complex Regional Pain Syndrome: Evaluation of the Potential Effects of Sound Feedback
Source: Front Hum Neurosci. 2017 Jul 27;11:379. doi: 10.3389/fnhum.2017.00379 (PMC5529353; doi:10.3389/fnhum.2017.00379)
Supplement: Supplementary file 7 [file Table7.DOCX]

**Table S7. Perceived body weight (in kilograms) for all conditions and for each participant according to the body distortion group.**

| **Distortion group** | **Participant id** | **Pre-test** | **Control condition** | **High frequency condition** | **Low frequency condition** |
| --- | --- | --- | --- | --- | --- |
| ‘Big’ | P04 | 108.50 | 107.00 | 103.50 | 98.00 |
|  | P10 | 54.00 | 73.00 | 73.50 | 75.50 |
|  | P07 | 78.50 | 96.50 | 93.00 | 88.50 |
| ‘Mixed’ | P03 | 85.50 | 94.50 | 88.50 | 93.50 |
|  | P08 | 97.50 | 103.50 | 100.50 | 92.50 |
| ‘Small’ | P01 | 75.00 | 69.50 | 63.50 | 67.00 |
| ‘Nothing’ | P05 | 75.50 | 79.50 | 77.50 | 77.00 |
|  | P12 | 35.50 | 52.50 | 48.50 | 52.00 |
|  | P09 | 110.50 | 97.00 | 98.50 | 93.00 |
|  | P11 | 55.50 | 58.50 | 73.00 | 69.00 |
|  | P06 | 33.50 | 51.00 | 51.00 | 59.50 |
|  | P02 | 67.00 | 60.50 | 69.50 | 73.00 |
