# Supplementary material for: Bodily Sensory Inputs and Anomalous Bodily Experiences in Complex Regional Pain Syndrome: Evaluation of the Potential Effects of Sound Feedback
Source: Front Hum Neurosci. 2017 Jul 27;11:379. doi: 10.3389/fnhum.2017.00379 (PMC5529353; doi:10.3389/fnhum.2017.00379)
Supplement: Supplementary file 8 [file Table8.DOCX]

**Table S8. SF-MPQ score for sensory descriptors, affective descriptors and total descriptors, for all conditions and for each participant according to the body disturbance group.** The SF-MPQ scores correspond to the sum of the intensity rank values of the words chosen for sensory, affective and total descriptors.

|  |  | **Pre-test** | | | **Control condition** | | | **High frequency condition** | | | **Low frequency condition** | | |
| --- | --- | --- | --- | --- | --- | --- | --- | --- | --- | --- | --- | --- | --- |
| **Distortion group** | **P Id** | **Sens.** | **Affec.** | **Total** | **Sens.** | **Affec.** | **Total** | **Sens.** | **Affec.** | **Total** | **Sens.** | **Affec.** | **Total** |
| ‘Big’ | P04 | 9 | 0 | 18 | 10 | 0 | 10 | 8 | 0 | 8 | 10 | 0 | 10 |
|  | P10 | 8 | 0 | 16 | 14 | 3 | 14 | 22 | 5 | 22 | 10 | 4 | 10 |
|  | P07 | 10 | 0 | 20 | 10 | 0 | 10 | 13 | 0 | 13 | 13 | 0 | 13 |
| ‘Mixed’ | P03 | 19 | 2 | 38 | 15 | 0 | 15 | 14 | 0 | 14 | 14 | 0 | 14 |
|  | P08 | 20 | 10 | 40 | 20 | 8 | 20 | 0 | 0 | 0 | 20 | 8 | 20 |
| ‘Small’ | P01 | 14 | 3 | 28 | 11 | 0 | 11 | 16 | 2 | 16 | 11 | 0 | 11 |
| ‘Nothing’ | P05 | 14 | 2 | 28 | 14 | 0 | 14 | 13 | 2 | 13 | 14 | 0 | 14 |
|  | P12 | 13 | 8 | 26 | 9 | 5 | 9 | 16 | 5 | 16 | 17 | 5 | 17 |
|  | P09 | 28 | 8 | 56 | 30 | 11 | 30 | 33 | 11 | 33 | 33 | 11 | 33 |
|  | P11 | 24 | 10 | 48 | 21 | 2 | 21 | 21 | 2 | 21 | 21 | 3 | 21 |
|  | P06 | 1 | 0 | 2 | 2 | 0 | 2 | 4 | 0 | 4 | 4 | 0 | 4 |
|  | P02 | 13 | 3 | 26 | 12 | 2 | 12 | 7 | 1 | 7 | 14 | 3 | 14 |
