# Supplementary material for: Bodily Sensory Inputs and Anomalous Bodily Experiences in Complex Regional Pain Syndrome: Evaluation of the Potential Effects of Sound Feedback
Source: Front Hum Neurosci. 2017 Jul 27;11:379. doi: 10.3389/fnhum.2017.00379 (PMC5529353; doi:10.3389/fnhum.2017.00379)
Supplement: Supplementary file 9 [file Table9.DOCX]

**Table S9. PPI score and VAS pain score for all conditions and for each participant according to the body disturbance group.** The PPI (Present Pain Intensity) index is a pain score ranging from 0 (no pain) to 5 (excruciating). The VAS pain score is a value between 0 and 10 cm, corresponding to a visual analogue rating scale.

|  |  | **Pre-test** | | **Control condition** | | **High frequency condition** | | **Low frequency condition** | |
| --- | --- | --- | --- | --- | --- | --- | --- | --- | --- |
| **Distortion group** | **P Id** | **PPI** | **VAS** | **PPI** | **VAS** | **PPI** | **VAS** | **PPI** | **VAS** |
| ‘Big’ | P04 | 2 | 6.50 | 3 | 7.60 | 3 | 7.60 | 2 | 5.00 |
|  | P10 | 1 | 2.75 | 3 | 6.95 | 4 | 7.70 | 3 | 6.20 |
|  | P07 | 2 | 7.60 | 2 | 6.50 | 3 | 8.50 | 2 | 8.80 |
| ‘Mixed’ | P03 | 3 | 6.75 | 2 | 6.85 | 2 | 7.25 | 2 | 7.30 |
|  | P08 | 4 | 6.90 | 4 | 8.15 | 4 | 8.05 | 4 | 7.85 |
| ‘Small’ | P01 | 2 | 6.25 | 2 | 3.70 | 2 | 7.45 | 1 | 3.05 |
| ‘Nothing’ | P05 | 3 | 6.00 | 3 | 6.85 | 3 | 6.30 | 2 | 6.35 |
|  | P12 | 4 | 6.35 | 3 | 8.35 | 3 | 8.65 | 3 | 9.00 |
|  | P09 | 4 | 6.75 | 5 | 9.65 | 5 | 9.25 | 4 | 8.35 |
|  | P11 | 2 | 8.45 | 3 | 7.60 | 4 | 7.80 | 4 | 7.90 |
|  | P06 | 2 | 3.90 | 2 | 4.25 | 2 | 5.75 | 2 | 4.85 |
|  | P02 | 4 | 6.50 | 4 | 6.60 | 3 | 5.30 | 3 | 5.30 |
