# Supplementary material for: Bodily Sensory Inputs and Anomalous Bodily Experiences in Complex Regional Pain Syndrome: Evaluation of the Potential Effects of Sound Feedback
Source: Front Hum Neurosci. 2017 Jul 27;11:379. doi: 10.3389/fnhum.2017.00379 (PMC5529353; doi:10.3389/fnhum.2017.00379)
Supplement: Supplementary file 10 [file Table10.DOCX]

**Table S10. Bath CRPS Body perception Disturbance questionnaire data for all conditions and for each participant according to body disturbance group.** The first four items are 11-level Likert items and the rest are frequency data. For the item “Part detached” the scale ranges from “very much a part” (0) to “completely detached” (11); for the item “Position unawareness” the scale ranges from “very aware” (0) to “completely unaware” (11); for the item “No attention to limb” the scale ranges from “full attention” (0) to “no attention” (11); for the item “Negative feelings” the scale ranges from “strongly positive” (0) to “strongly negative” (11).

| Condition | Distortion group | P Id | Part detached | Position unawareness | No attention to limb | Negative feelings | Change Size | Change Temperature | Change Pressure | Change Weight |
| --- | --- | --- | --- | --- | --- | --- | --- | --- | --- | --- |
| Pre-test | ‘Big’ | P04 | 5 | 3 | 5 | 5 | 0 | 1 | 1 | 1 |
|  |  | P10 | 4 | 4 | 1 | 8 | 1 | 1 | 1 | 1 |
|  |  | P07 | 7 | 7 | 7 | 8 | 1 | 1 | 1 | 1 |
|  | ‘Mixed’ | P03 | 10 | 10 | 9 | 10 | 1 | 1 | 1 | 1 |
|  |  | P08 | 10 | 3 | 10 | 10 | 1 | 1 | 1 | 1 |
|  | ‘Small’ | P01 | 3 | 8 | 2 | 5 | 1 | 1 | 1 | 1 |
|  | ‘Nothing’ | P05 | 7 | 7 | 5 | 8 | 1 | 1 | 1 | 1 |
|  |  | P12 | 8 | 8 | 10 | 10 | 1 | 1 | 1 | 1 |
|  |  | P09 | 8 | 8 | 2 | 9 | 1 | 1 | 1 | 1 |
|  |  | P11 | 10 | 9 | 10 | 10 | 1 | 1 | 1 | 1 |
|  |  | P06 | 4 | 3 | 8 | 6 | 1 | 1 | 1 | 1 |
|  |  | P02 | 7 | 8 | 5 | 6 | 1 | 1 | 1 | 1 |
| Control | ‘Big’ | P04 | 2 | 5 | 5 | 5 | 0 | 1 | 1 | 1 |
|  |  | P10 | 7 | 8 | 1 | 8 | 1 | 1 | 1 | 1 |
|  |  | P07 | 7 | 8 | 7 | 7 | 1 | 1 | 1 | 1 |
|  | ‘Mixed’ | P03 | 9 | 10 | 10 | 10 | 1 | 1 | 1 | 1 |
|  |  | P08 | 10 | 3 | 10 | 10 | 1 | 1 | 1 | 1 |
|  | ‘Small’ | P01 | 7 | 7 | 7 | 3 | 0 | 1 | 0 | 1 |
|  | ‘Nothing’ | P05 | 6 | 4 | 5 | 6 | 1 | 1 | 1 | 1 |
|  |  | P12 | 3 | 3 | 3 | 10 | 1 | 1 | 1 | 1 |
|  |  | P09 | 8 | 8 | 8 | 9 | 1 | 1 | 1 | 1 |
|  |  | P11 | 10 | 9 | 10 | 10 | 1 | 1 | 0 | 1 |
|  |  | P06 | 8 | 8 | 7 | 8 | 0 | 0 | 1 | 1 |
|  |  | P02 | 4 | 5 | 4 | 5 | 1 | 1 | 1 | 1 |
| High frequency | ‘Big’ | P04 | 3 | 5 | 5 | 3 | 1 | 1 | 1 | 1 |
|  |  | P10 | 9 | 9 | 7 | 7 | 1 | 1 | 1 | 1 |
|  |  | P07 | 8 | 8 | 7 | 8 | 1 | 1 | 1 | 1 |
|  | ‘Mixed’ | P03 | 9 | 10 | 10 | 10 | 1 | 1 | 1 | 1 |
|  |  | P08 | 10 | 3 | 10 | 10 | 1 | 1 | 1 | 1 |
|  | ‘Small’ | P01 | 4 | 0 | 2 | 5 | 1 | 1 | 1 | 1 |
|  | ‘Nothing’ | P05 | 6 | 4 | 4 | 7 | 1 | 1 | 1 | 1 |
|  |  | P12 | 3 | 3 | 3 | 10 | 1 | 1 | 1 | 1 |
|  |  | P09 | 4 | 4 | 3 | 4 | 1 | 1 | 1 | 1 |
|  |  | P11 | 10 | 10 | 10 | 10 | 1 | 1 | 0 | 1 |
|  |  | P06 | 9 | 9 | 8 | 8 | 0 | 1 | 1 | 1 |
|  |  | P02 | 4 | 5 | 4 | 4 | 1 | 1 | 1 | 1 |
| Low frequency | ‘Big’ | P04 | 4 | 5 | 5 | 3 | 1 | 1 | 1 | 1 |
|  |  | P10 | 8 | 8 | 1 | 8 | 1 | 1 | 1 | 1 |
|  |  | P07 | 7 | 7 | 7 | 8 | 1 | 1 | 1 | 1 |
|  | ‘Mixed’ | P03 | 10 | 10 | 10 | 10 | 1 | 1 | 1 | 1 |
|  |  | P08 | 10 | 3 | 10 | 10 | 1 | 1 | 1 | 1 |
|  | ‘Small’ | P01 | 3 | 3 | 4 | 5 | 1 | 1 | 1 | 1 |
|  | ‘Nothing’ | P05 | 5 | 5 | 4 | 7 | 1 | 1 | 1 | 1 |
|  |  | P12 | 10 | 9 | 10 | 10 | 1 | 1 | 1 | 1 |
|  |  | P09 | 9 | 9 | 9 | 8 | 1 | 1 | 1 | 1 |
|  |  | P11 | 10 | 9 | 10 | 10 | 1 | 1 | 0 | 1 |
|  |  | P06 | 8 | 8 | 7 | 7 | 0 | 1 | 0 | 1 |
|  |  | P02 | 6 | 6 | 6 | 6 | 1 | 1 | 1 | 1 |
