# Supplementary material for: Bodily Sensory Inputs and Anomalous Bodily Experiences in Complex Regional Pain Syndrome: Evaluation of the Potential Effects of Sound Feedback
Source: Front Hum Neurosci. 2017 Jul 27;11:379. doi: 10.3389/fnhum.2017.00379 (PMC5529353; doi:10.3389/fnhum.2017.00379)
Supplement: Supplementary file 11 [file Table11.DOCX]

**Table S11. Body feelings data for all conditions and for each participant according to body disturbance group.** The values correspond to 7-level Likert items. For the item “Speed” the scale ranges from “slow” (1) to “quick” (7); for the item “Weight” the scale ranges from “light” (1) to “heavy” (7); for the item “Strength” the scale ranges from “weak” (1) to “strong” (7); for the item “Straight” the scale ranges from “crouched, stoop” (1) to “elongated, extended” (7). For the remaining items (“Agency”, “Vividness”, “Surprise”, “Feet localization”), the scale indicates the level of agreement with the statement, ranging from “I strongly disagree” (1) to “I strongly agree” (7).

| **Condition** | **Distortion group** | **P Id** | **Speed** | **Weight** | **Strength** | **Straight** | **Agency** | **Vividness** | **Surprise** | **Feet localization** |
| --- | --- | --- | --- | --- | --- | --- | --- | --- | --- | --- |
| Pre-test | ‘Big’ | P04 | 3 | 6 | 4 | 4 | 5 | 6 | 4 | 3 |
|  |  | P10 | 2 | 6 | 2 | 2 | 3 | 3 | 2 | 3 |
|  |  | P07 | 3 | 4 | 4 | 3 | 3 | 3 | 5 | 4 |
|  | ‘Mixed’ | P03 | 2 | 7 | 3 | 3 | - | - | - | 1 |
|  |  | P08 | 1 | 7 | 1 | 3 | 4 | 1 | 7 | 5 |
|  | ‘Small’ | P01 | 2 | 6 | 4 | 3 | - | - | - | 2 |
|  | ‘Nothing’ | P05 | 1 | 4 | 2 | 3 | 3 | 3 | 5 | 2 |
|  |  | P12 | 4 | 4 | 4 | 4 | 4 | 4 | 5 | 5 |
|  |  | P09 | 4 | 4 | 5 | 5 | 4 | 4 | 4 | 2 |
|  |  | P11 | 1 | 6 | 3 | 2 | 1 | 5 | 5 | 2 |
|  |  | P06 | 5 | 4 | 5 | 5 | 4 | 4 | 4 | 4 |
|  |  | P02 | 2 | 7 | 2 | 1 | - | - | - | 1 |
| Control | ‘Big’ | P04 | 3 | 6 | 4 | 4 | 4 | 5 | 4 | 4 |
|  |  | P10 | 2 | 6 | 3 | 2 | 4 | 3 | 4 | 2 |
|  |  | P07 | 4 | 2 | 5 | 6 | 6 | 5 | 5 | 6 |
|  | ‘Mixed’ | P03 | 4 | 5 | 4 | 4 | 4 | 3 | 4 | 1 |
|  |  | P08 | 1 | 7 | 1 | 3 | 1 | 1 | 7 | 3 |
|  | ‘Small’ | P01 | 4 | 3 | 4 | 5 | 2 | 2 | 6 | 5 |
|  | ‘Nothing’ | P05 | 2 | 6 | 2 | 2 | 3 | 4 | 5 | 3 |
|  |  | P12 | 6 | 6 | 2 | 2 | 6 | 2 | 6 | 6 |
|  |  | P09 | 3 | 6 | 3 | 4 | 3 | 4 | 4 | 2 |
|  |  | P11 | 1 | 5 | 1 | 2 | 1 | 1 | 2 | 1 |
|  |  | P06 | 2 | 6 | 5 | 5 | 4 | 6 | 7 | 4 |
|  |  | P02 | 2 | 6 | 2 | 1 | 2 | 5 | 3 | 5 |
| High frequency | ‘Big’ | P04 | 6 | 3 | 5 | 6 | 6 | 2 | 6 | 6 |
|  |  | P10 | 3 | 7 | 1 | 1 | 1 | 2 | 2 | 1 |
|  |  | P07 | 4 | 3 | 4 | 5 | 3 | 4 | 5 | 5 |
|  | ‘Mixed’ | P03 | 4 | 5 | 3 | 4 | 7 | 3 | 4 | 3 |
|  |  | P08 | 1 | 7 | 1 | 3 | 1 | 1 | 7 | 3 |
|  | ‘Small’ | P01 | 3 | 5 | 2 | 2 | 6 | 4 | 2 | 7 |
|  | ‘Nothing’ | P05 | 2 | 5 | 2 | 3 | 5 | 3 | 5 | 5 |
|  |  | P12 | 3 | 7 | 2 | 2 | 5 | 2 | 2 | 5 |
|  |  | P09 | 5 | 6 | 4 | 4 | 6 | 5 | 6 | 5 |
|  |  | P11 | 1 | 3 | 2 | 2 | 1 | 5 | 4 | 1 |
|  |  | P06 | 3 | 5 | 4 | 5 | 3 | 6 | 5 | 5 |
|  |  | P02 | 3 | 5 | 2 | 2 | 5 | 5 | 5 | 4 |
| Low frequency | ‘Big’ | P04 | 5 | 4 | 4 | 6 | 6 | 3 | 6 | 5 |
|  |  | P10 | 2 | 6 | 2 | 2 | 2 | 6 | 2 | 2 |
|  |  | P07 | 5 | 2 | 5 | 6 | 6 | 6 | 5 | 6 |
|  | ‘Mixed’ | P03 | 4 | 4 | 4 | 3 | 7 | 4 | 4 | 6 |
|  |  | P08 | 1 | 7 | 1 | 3 | 7 | 1 | 7 | 3 |
|  | ‘Small’ | P01 | 5 | 3 | 4 | 6 | 6 | 2 | 5 | 6 |
|  | ‘Nothing’ | P05 | 3 | 5 | 3 | 3 | 5 | 4 | 5 | 5 |
|  |  | P12 | 2 | 6 | 2 | 2 | 3 | 2 | 3 | 5 |
|  |  | P09 | 6 | 6 | 4 | 5 | 6 | 3 | 6 | 3 |
|  |  | P11 | 1 | 6 | 2 | 2 | 1 | 5 | 5 | 2 |
|  |  | P06 | 3 | 6 | 4 | 5 | 5 | 6 | 5 | 4 |
|  |  | P02 | 2 | 6 | 2 | 1 | 5 | 3 | 4 | 3 |
