# Supplementary material for: Bodily Sensory Inputs and Anomalous Bodily Experiences in Complex Regional Pain Syndrome: Evaluation of the Potential Effects of Sound Feedback
Source: Front Hum Neurosci. 2017 Jul 27;11:379. doi: 10.3389/fnhum.2017.00379 (PMC5529353; doi:10.3389/fnhum.2017.00379)
Supplement: Supplementary file 12 [file Table12.DOCX]

**Table S12. Emotional valence (Val), Arousal (Aro) and Dominance (Dom) for all three sound conditions for each participant, according to their body disturbance group.** The values correspond to 9-level Likert items. Valence, Arousal and Dominance ratings refer to the 9-item graphic scales of the Self-assessment Manikin questionnaire.

|  |  | **Pre-test** | | | **Control condition** | | | **High frequency condition** | | | **Low frequency condition** | | |
| --- | --- | --- | --- | --- | --- | --- | --- | --- | --- | --- | --- | --- | --- |
| **Distortion group** | **P Id** | **Val** | **Aro** | **Dom** | **Val** | **Aro** | **Dom** | **Val** | **Aro** | **Dom** | **Val** | **Aro** | **Dom** |
| ‘Big’ | P04 | 6 | 5 | 5 | 5 | 5 | 5 | 6 | 3 | 6 | 7 | 6 | 7 |
|  | P10 | 5 | 6 | 3 | 3 | 6 | 3 | 3 | 7 | 2 | 3 | 7 | 3 |
|  | P07 | 5 | 6 | 3 | 8 | 7 | 7 | 7 | 7 | 6 | 7 | 7 | 7 |
| ‘Mixed’ | P03 | 7 | 3 | 5 | 6 | 3 | 5 | 7 | 2 | 5 | 7 | 2 | 5 |
|  | P08 | 2 | 8 | 2 | 1 | 8 | 1 | 1 | 8 | 1 | 1 | 8 | 1 |
| ‘Small’ | P01 | 7 | 5 | 5 | 7 | 7 | 6 | 7 | 6 | 4 | 7 | 7 | 6 |
| ‘Nothing’ | P05 | 5 | 5 | 2 | 6 | 4 | 6 | 6 | 6 | 6 | 6 | 6 | 6 |
|  | P12 | 5 | 5 | 5 | 7 | 7 | 6 | 6 | 7 | 3 | 7 | 4 | 4 |
|  | P09 | 9 | 7 | 9 | 7 | 6 | 7 | 8 | 6 | 7 | 7 | 7 | 8 |
|  | P11 | 2 | 3 | 1 | 2 | 6 | 2 | 2 | 3 | 2 | 2 | 4 | 2 |
|  | P06 | 8 | 6 | 5 | 5 | 6 | 5 | 5 | 5 | 5 | 6 | 7 | 5 |
|  | P02 | 7 | 6 | 4 | 5 | 5 | 5 | 7 | 4 | 5 | 6 | 6 | 5 |
