# Supplementary material for: Bodily Sensory Inputs and Anomalous Bodily Experiences in Complex Regional Pain Syndrome: Evaluation of the Potential Effects of Sound Feedback
Source: Front Hum Neurosci. 2017 Jul 27;11:379. doi: 10.3389/fnhum.2017.00379 (PMC5529353; doi:10.3389/fnhum.2017.00379)
Supplement: Supplementary file 13 [file Image1.PDF]

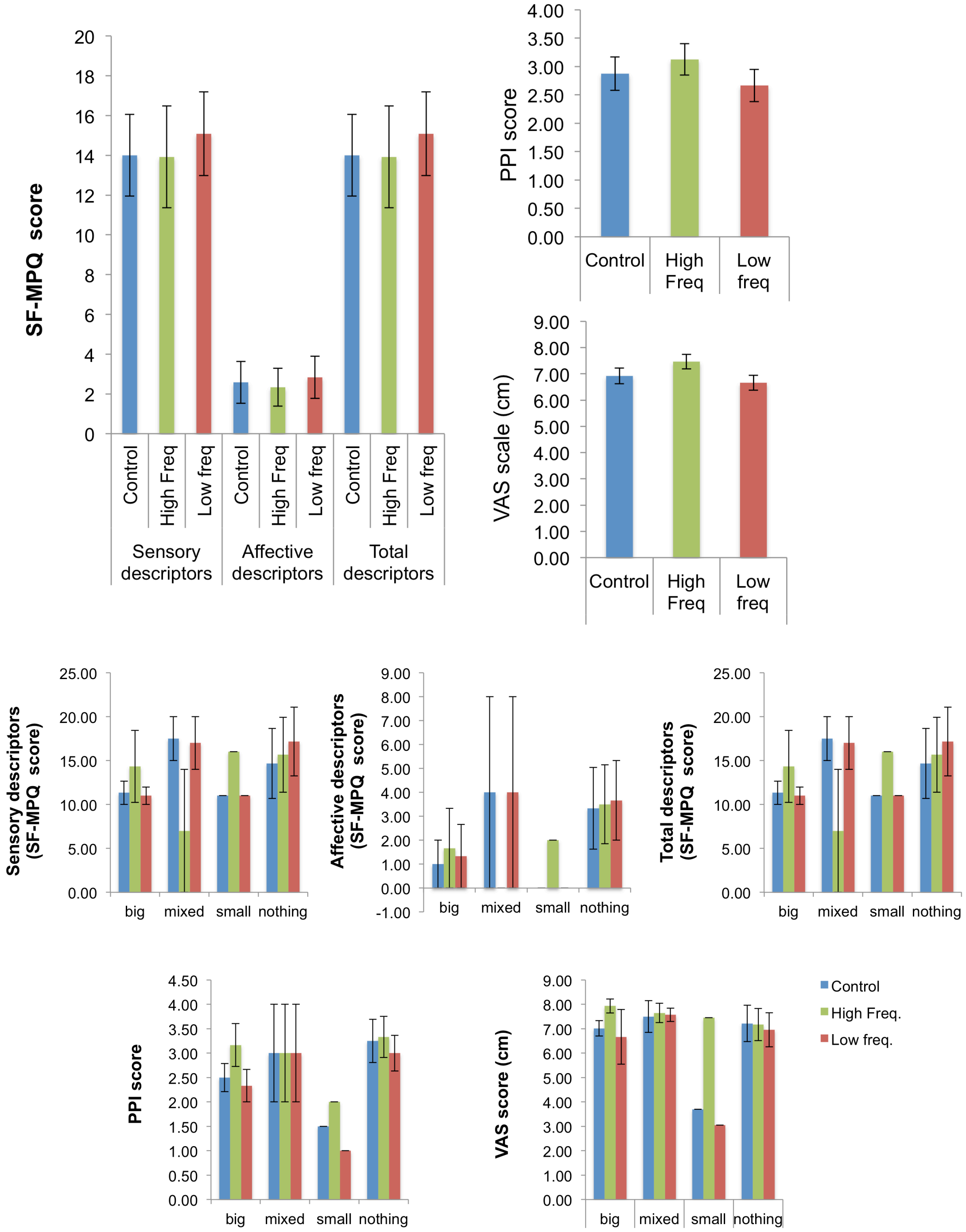

Figure S1. Mean SF-MPQ score, PPI score and VAS pain score (±SE) for each body distortion group: 'Big' (N=3), 'Mixed' (N=2), 'Small' (N=1), 'Nothing' (N=6).
